# Operationalizing clinical artificial intelligence through secure FHIR pipelines: a zero-trust cloud reference architecture for EHR interoperability

**DOI:** 10.3389/fdgth.2026.1953969

**Published:** 2026-09-29

**Authors:** Femi Oke

**Affiliations:** School of Computing, Data, and Mathematical Sciences, College of Business, Health, and Community Programs, Western Illinois University, Macomb, IL, United States

**Keywords:** AI governance, clinical artificial intelligence, clinical decision support, electronic health records, evidence continuity, fast healthcare interoperability resources, MLOps, zero trust

## Abstract

Fast Healthcare Interoperability Resources (FHIR) standardizes clinical exchange but does not establish that a downstream artificial intelligence (AI) use remains authorized, semantically consistent, or accountable. This Hypothesis and Theory article proposes evidence-continuous boundary control: a material data, model, or workflow transition is governed by an applicable policy decision and linked to versioned evidence of its prerequisites, execution, and disposition. The framework arose from continuing healthcare-interoperability research and was formalized through standards analysis, targeted evidence synthesis, and explicit design inference. Documented revision searches distinguish external evidence from architectural decisions. Eight requirements are organized into five functional layers: clinical sources, an interoperability edge, a zero-trust control plane, a segregated cloud data and AI plane, and governance and monitoring. Applications are consumers, not a sixth layer. An illustrative adult readmission-risk workflow connects identity, policy, source versions, features, model release, scoring, presentation, and human action. Its symbolic FHIR R4 evidence linkage includes explicit R5 translation cautions and policy-aware authorization, including consent where applicable. Six propositions test incremental value against a well-run contemporary platform without removing its native safeguards; simulated ablations isolate particular links. Independent reference standards, denominators, support, counterevidence, and inconclusive conditions expose operational and human-factors trade-offs. A bounded synthetic-data companion implementation makes selected exchange controls inspectable; it does not implement the complete clinical-AI architecture or validate its propositions. No empirical architecture evaluation, patient dataset, clinical effectiveness, or independent expert validation is reported. Staged future studies must establish incremental benefit without disproportionate burden or inappropriate reliance.

## 1 Introduction

FHIR exchange, SMART authorization, population export, and workflow-triggered decision support provide important building blocks for clinical information systems (1–4). They do not, individually or merely by being present together, establish that a particular clinical AI output can be traced through an authorized and semantically coherent sequence of transformations. For example, an authorized source read could feed a feature definition that has changed, a model approved for another population, or a display associated with the wrong encounter. The relevant question is not whether an organization possesses access controls and logs, but whether those controls and records remain consistent across the boundaries that determine a specific use.

The proposed contribution is an explicit, testable contract between boundary enforcement and evidence continuity. The contract binds the identity and policy decision for a transition to the particular data, feature, model, release, scoring, and workflow artifacts involved. It also requires missing or incompatible prerequisites to be detectable rather than interpreted as successful completion. This is an author-derived integration of existing ideas, not the invention of zero trust, provenance, FHIR, or machine-learning operations (MLOps). Zero-trust guidance establishes identity- and resource-oriented access principles; cloud-native guidance extends these principles to service identities; AI risk guidance addresses lifecycle accountability beyond cybersecurity (5–7). This article asks whether linking their application across clinical AI boundaries offers incremental benefit over well-run local controls.

This article develops eight requirements, five functional layers, an asset-centered threat model, six propositions, and a readmission-risk illustration. The theoretical claim is conditional: with equivalent individual safeguards and adequately specified policies, checking linked prerequisites at material transitions should reduce cross-boundary inconsistencies and improve reconstruction without unacceptable denial, latency, privacy, or workflow burdens. A system with strong local controls may already achieve equivalent outcomes. Such equivalence would limit the framework's incremental contribution.

The complete clinical-AI architecture and its six propositions are not implemented or evaluated in this article. A bounded companion implementation from the same research program is described in Section 2.4 only to identify inspectable exchange mechanisms and their limits. The architecture walkthrough, requirements mapping, and readmission scenario are conceptual design products, not measurements of effectiveness, technical certification, expert validation, or evidence of patient benefit.

## 2 Conceptual development, evidence synthesis, and design inference

### 2.1 Research origin and development pathway

The architecture arose from a continuing research and design program. Its intellectual and technical foundation was the author's 2024 Western Illinois University graduate project, Secure Data Integration Pipeline Using Fast Healthcare Interoperability Resources (FHIR) Standards. That project addressed fragmented clinical information, insecure or inefficient interorganizational exchange, and limited patient control. Its planned work included requirements research, architecture, patient authorization, encryption, consent management, FHIR interoperability, implementation, testing, and validation. The academic-origin record and linked privacy-redacted proposal document this foundation, but do not establish that every file in the current implementation existed in 2024 (8).

The present theory extends this interoperability problem into the clinical AI lifecycle. Authorization and evidence must remain checkable across source retrieval, transformation, feature definition, model release, scoring, presentation, and human disposition. Requirements R1-R8, the five layers, evidence-continuous boundary control, and propositions P1-P6 formalize this design problem. Literature, standards, policy guidance, implementation studies, and competing architectures informed its refinement, scope, and comparative positioning. The source-to-design mapping was documented during revision; it records the reasoning supporting the framework rather than a contemporaneous account of every step in its earlier development.

A bounded implementation existed locally within this continuing research before the initial manuscript submission on 30 July 2026. Version v1.0.0 was publicly released on GitHub on 9 August 2026 for transparency and independent technical inspection (9). This is a dissemination date, not the implementation's creation date. The public release and Git history identify the published artifact, but do not reconstruct the complete earlier local development history or establish that all released code and tests predated submission. Neither the local implementation nor its later public release is used as empirical validation of the complete theory.

### 2.2 Evidence synthesis and revision-stage search

This is a conceptual synthesis, not a systematic or scoping review. A prospectively registered search protocol and a complete candidate-level screening ledger were not maintained during the earlier development phase. Original hit counts, exclusion counts, and exact historical search dates therefore cannot be reconstructed reliably. The original manuscript's 45 references provide a bibliographic baseline, not a screening denominator. The revision record contains 46 first-stage query strings and 26 follow-up queries, all dated 10 September 2026. These documented revision searches are reported separately from the earlier research history in Supplementary Tables S1A,B.

Retrieval used a general web-search interface and direct examination of official, publisher, and PubMed/PMC records. Queries addressed named FHIR and clinical deployment architectures, zero trust and usage control, provenance and assurance, appropriate reliance, version-sensitive standards claims, and the four titles suggested by Reviewer 2. Exact-title and DOI queries checked candidate records; versioned HL7 pages were examined for resource semantics and release differences. Supplementary Table S1 retains the literal queries, dates, interface, and recorded restrictions. No native PubMed or subscription-database search, exhaustive retrieval, dual screening, or formal risk-of-bias assessment was performed. Web rankings and page contents may change when queries are repeated.

Eight further queries on 10 September 2026 checked Frontiers requirements, the ISAS presentation record, and the four suggested publications. Version-specific repository and official program records were also retrieved directly. These checks are recorded in Supplementary Table S1C. Final editorial and source checks on 11 September 2026 are recorded separately in S1D and did not add scientific sources to the inventory. Supplementary Tables S6, S7 identify the material accessible for each source and the reasons for its inclusion or exclusion. Abstract or metadata access is not described as full-text appraisal.

### 2.3 Source selection and explicit design inference

Sources were retained when accessible content directly informed a control objective, named architectural comparison, boundary assumption, threat mechanism, human-factors consideration, or prospective evaluation criterion. Official specifications governed field semantics and release-sensitive claims; policy guidance bounded legal descriptions; implementation publications established capabilities reported for named systems; empirical studies informed observations within their own settings; and reviews or theoretical work supplied context and mechanisms. Task-specific XAI studies were retained only when relevant to these purposes. Supplementary Table S6 records individual scope and access assessments for all four reviewer suggestions. A feature not described in a publication is treated as unknown, not absent.

The documented inventory contains 56 distinct records, of which 53 are cited in the main bibliography and three task-specific XAI papers are assessed but not retained. The 53 cited records comprise 50 scientific, standards, or guidance sources and three provenance records covering the academic origin, versioned companion software, and prior conference abstract. These are inventory and disposition counts, not estimates of all retrieved hits or the number screened during the original research. The provenance records document the author's work; they do not provide independent support for the propositions. Supplementary Tables S6, S7 record each disposition.

Source records distinguish S (published specification), G (guidance), G/L (official explanation of legal requirements), E (empirical study), R (review), and T (theoretical or methodological contribution); combinations are allowed. A published FHIR resource is not automatically normative: maturity and trial-use status remain resource- and release-specific. PROV-DM is a W3C Recommendation with normative definitions, whereas the worked R4 Consent, Provenance, AuditEvent, and RiskAssessment resources retain trial-use status (10–14). Standards specify representations or interfaces, guidance motivates objectives, and empirical studies report observations in their own settings. None supplies empirical support for this complete architecture. Decisions about the five-layer decomposition, the material-transition contract, and the six propositions are identified as architectural inferences rather than findings extracted from a source.

The source-to-design mapping was documented during revision in four steps. First, a source statement was paraphrased as a control objective. The objective codes are CO1 semantic integrity, CO2 attributable identity, CO3 lawful and purpose-bounded use, CO4 isolation and containment, CO5 artifact and evidence integrity, CO6 accountable model change, CO7 detection and appropriate human response, and CO8 bounded deployment. Second, an objective justified a requirement when its omission exposed a material failure and an observable control responsibility could be assigned. Source authority, scope, applicability, and the consequences of omission took precedence over citation frequency. Third, responsibilities were grouped by authority and change cadence. Fourth, each proposed mechanism was paired with an independent reference standard and a matched comparison. These rules make the design reasoning inspectable; they do not reconstruct an original coding protocol.

Supplementary Table S2 links each source to its control objective, the author's design inference, the relevant requirement, the responsible layer or control point, and a proposition or evaluation criterion. Supplementary Table S7 records the individual source anchor, access scope, and retention rationale. For example, FHIR version references inform CO1 and CO5 and requirements R1 and R5 at L1, L2, and L4. Identity guidance informs CO2 and R2 at L3, while model documentation informs CO6 and R6 at L4 and L5. Requiring a scoring checkpoint to verify their joint compatibility is the proposed inference, assessed principally by P1 and P4. No individual source establishes that joint effect. Cross-cutting requirements may map to several layers and propositions; alternative groupings remain acceptable when they preserve the same responsibilities.

The synthesis was conducted by one author without independent human screening, adjudication, coding-reliability assessment, or expert validation. Selection bias, incomplete coverage, and dependence on accessible reports remain limitations. The documented mappings provide a rationale for the design, not evidence that the complete architecture has been validated.

### 2.4 Bounded companion reference implementation

The companion Consent-Aware FHIR Exchange Prototype, v1.0.0, is an executable, synthetic-data-only artifact from the same research program (9). Its documented stack is HAPI FHIR R4, Spring Boot, PostgreSQL, and React. Within a bounded patient-directed exchange scenario, it implements consent approval, denial, expiry and revocation; organization-aware authorization; purpose-of-use and resource-type scope enforcement; FHIR Bundle generation and validation; protected package handling and integrity verification; and correlated AuditEvent and Provenance records. Its documented negative paths include denied, expired, revoked, unauthenticated, wrong-organization, out-of-scope, invalid-FHIR, and altered-package cases. The repository provides source, execution instructions, and technical test materials for inspection; test counts are not imported as findings of this paper.

The correspondence is partial and functional, not a deployment of five complete layers. FHIR generation/validation illustrates part of R1 at L2; authenticated organizational scope and bounded consent checks illustrate parts of R2/R3 at L3; protected packages illustrate part of R5; and correlated exchange evidence informs selected R7/L5 concerns. These mappings do not establish enterprise workload federation, operational/analytic isolation under R4, governed model execution under R6, hybrid rollout under R8, or end-to-end scoring-to-human-action evidence continuity. In particular, the artifact does not implement the complete feature/model-release, RiskAssessment scoring, workflow-presentation, and clinician-action chain proposed for the readmission illustration. It cannot validate P1-P6.

The implementation has no clinical deployment or clinical validation and is not approved for real patient information. Its scope does not include minors, guardianship or delegated/surrogate consent, emergency or break-glass workflows, jurisdiction-specific legal adjudication, or comprehensive sensitivity segmentation. Its patient-directed consent workflow is not a claim that consent is the only lawful basis for healthcare use; Section 7 describes the broader authorization theory, including pathways that this artifact does not implement. The repository also identifies prototype rather than production identity/key custody and tamper-evident rather than independently immutable logging. Public inspectability is not a certification of security, legal compliance, clinical effectiveness, or complete architectural feasibility.

## 3 Related work and comparative positioning

### 3.1 Named FHIR, deployment, and hospital platforms

FHIR-DHP provides a concrete harmonization pipeline with hospital querying, FHIR mapping, syntactic checking, a patient-model database, and AI-oriented export, evaluated in its reported development context (15). DEPLOYR addresses the difficult transition from retrospective development to live electronic medical record integration through development, serving, and monitoring applications; its reported functions include real-time access and prospective deployment (16). These are substantive predecessors. The present comparison asks whether an implementation makes a particular policy decision, source version, model release, and clinical presentation mutually checkable across transitions. The absence of that explicit contract from a report does not establish its absence from the deployed system.

Secure-healthcare predecessors must also be credited. FHIRChain combines FHIR-oriented clinical sharing with identity and blockchain-linked access mechanisms (17). Its reported sharing design is a relevant secure-data foundation, not an obviously insecure comparator. Spadacini's Clinical MLOps framework already describes runtime consent/purpose checks, versioned lineage and inference records, clinical outcome linkage, and human oversight (18). Those overlaps materially narrow the present claim. The accessible preprint version was examined; its linked journal version was not retrievable, so equivalence between versions and its empirical or legal claims are not assumed. Our proposed increment is a specified episode-level prerequisite and expected-event contract spanning these functions, not the discovery that clinical MLOps needs governance.

Maimaitiaili and colleagues synthesize hospital AI-platform architectures into infrastructure, data, algorithm, application, and security/compliance functions (19). Their five-layer arrangement differs from this paper: applications are a layer there, whereas here they consume outputs from an operating architecture with separately identified runtime policy and governance responsibilities. Neither number of layers nor the placement of a box establishes superiority. The useful comparison is how responsibilities, interfaces, and evidence obligations are allocated. Table 1 compares these named approaches with the present contract and states the limits of what can be inferred from their publications.

**Table 1:** Named comparators and the specific question posed by the boundary-control contract. “Not established by the report” is not a claim that a feature is absent from the underlying system.

Named approach	Reported contribution	Relationship to the proposed contract
FHIR-DHP; FHIRChain (15, 17)	Clinical harmonization/AI export; secure FHIR-oriented sharing with identity/access mechanisms.	Established foundations, not weak baselines. Compare actual policy-to-transformation and score-to-use linkage; unreported details are unknown.
DEPLOYR; Clinical MLOps (16, 18)	Clinical deployment/monitoring; runtime purpose checks, versioned inference lineage, outcome linkage and human oversight.	Substantial overlap. Test incremental prerequisite enforcement and expected-event reconciliation; retain native safeguards. Clinical MLOps comparison uses its accessible preprint.
Hospital AI-platform review (19)	Infrastructure, data, algorithm, application and security/compliance layers.	Same broad responsibilities, different grouping. Neither layer count establishes necessity, sufficiency or superiority.
NIST zero trust; UCONABC (5, 6, 24)	Identity/resource access and service identities; ongoing authorizations, obligations, conditions and mutable attributes.	Continuous policy control is prior art. The proposed specialization binds clinical purpose and typed episode prerequisites to these controls.
Log management; FHIR AuditEvent; PROV-DM; in-toto (10, 13, 23, 25)	Event recording/reconciliation, general provenance, and signed supply-chain step/artifact verification.	Logging, lineage and cross-stage integrity are not new. An equivalent contract implemented by these mechanisms is a valid rival design.
AMLAS (26)	Lifecycle safety arguments with supporting assurance evidence.	Runtime records may support an assurance case; neither a linked graph nor an architecture diagram establishes that case.
SMART, CDS Hooks and clinical reasoning standards (2, 4, 22)	Authorization and interoperable workflow interfaces.	Supply mechanisms rather than guarantees of appropriate use; encounter binding, presentation evidence and human action remain separately testable.

FHIR implementation reviews provide context for semantic, integration, and operational challenges (20, 21). Standards tutorials explain how SMART, CDS Hooks, and clinical reasoning mechanisms fit together (22). This paper builds on those interfaces rather than proposing a replacement interoperability stack. It does not claim that all FHIR pipelines lack model governance or that all clinical MLOps platforms omit privacy controls.

### 3.2 Zero trust, audit, provenance, and assurance are complementary

NIST zero trust and its cloud-native extension motivate independent workload identity and resource-specific policy enforcement (5, 6). They do not decide whether a readmission model is clinically appropriate. NIST log-management guidance concerns collection and management of security events (23), while W3C PROV-DM supplies a vocabulary for entities, activities, agents, and their relationships (10). The proposed contract uses event and lineage records as dependencies of a material transition, including explicit reconciliation of expected and observed events. It is narrower than a general provenance model and stronger than merely collecting records without checking their cross-boundary consistency. FHIR AuditEvent itself anticipates records from multiple actors and the use of missing or conflicting records to investigate safeguards (13). The distinction proposed here is therefore not multi-actor logging, but an explicit episode-level dependency contract spanning policy, feature/model release, and clinical disposition, with relevant dependencies also used in enforcement.

Continuous use control and integrity-linked process steps also predate this framework. UCONABC explicitly models authorizations, obligations, conditions, mutable attributes, and ongoing usage decisions (24). The in-toto framework verifies signed evidence of supply-chain steps and artifact relationships (25). Accordingly, neither continuous policy evaluation nor cross-stage attestation is claimed as new. Here they motivate a clinical specialization: a governed data/model transition must bind its lawful purpose, patient/encounter scope, source and feature versions, release, output, presentation, and disposition to independently specified event expectations. That specialization is only an incremental research claim; an existing usage-control, provenance, or attestation implementation that satisfies the same contract is an equivalent design, not an excluded rival.

AMLAS addresses structured safety assurance across a machine-learning lifecycle (26). An assurance case relates claims, arguments, assumptions, and evidence; an operational evidence graph records particular artifacts and actions. The latter may supply evidence to the former but cannot establish that the argument is sound or that a system is safe. Similarly, distributed trace identifiers support correlation but do not authenticate an event's truth or prove lawful use (27). A contemporary system that already enforces an equivalent linkage contract is an overlapping implementation, not a counterexample to be dismissed by terminology.

### 3.3 Model governance and appropriate reliance

Model cards document intended use and evaluation context (28), and clinical-ML lifecycle work and FUTURE-AI extend attention to deployment conditions, oversight, and ongoing risk management (29, 30). These governance concerns are not consequences of network security. The distinction matters: a correctly authenticated service can run an approved but unsuitable model, and an intact evidence chain can document an unsafe decision perfectly.

Appropriate reliance, rather than maximized trust, is the human-factors objective (31). Gaube et al. experimentally examined responses to inaccurate advice, illustrating that advice can impair decisions; that study does not validate this architecture or isolate the effect of a polished provenance panel (32). Wani et al. review explainability techniques and challenges in healthcare/IoMT (33). We use that review for its XAI context; the distinction between explanatory content and authorization/provenance evidence is our architectural interpretation. An explanation can be understandable while a use is unauthorized, and a fully traceable output can still be clinically wrong. Whether detailed-looking context increases unwarranted reliance is a plausible countermechanism to test in P6, not a demonstrated benefit or harm of this framework.

## 4 Theory: evidence-continuous boundary control

### 4.1 Definitions and scope of the claim

A material transition is an event that changes the principal or delegation, permitted purpose or scope, data meaning or version, feature definition, model/release state, destination, or clinical disposition in a way that could alter access, interpretation, or accountability. Examples are protected retrieval, export, transformation, feature generation, release, scoring, presentation, and recording a human action. An internal computation that changes none of these properties need not become a separate authorization transaction. Materiality must be specified in the local use-case state model before evaluation, not retrospectively expanded to make recorded events appear complete.

Evidence continuity is the ability to follow the required typed and versioned relationships between material events and artifacts, including unsuccessful or exceptional paths, and to distinguish a complete path from an unresolved one. It is not the presence of many logs, a common timestamp, or a single correlation identifier. A material event needs identifiable predecessors, an applicable policy basis, an execution disposition, and, when expected, a successor acknowledgment. A human response may legitimately be pending, declined, or not captured; those states must not be converted into acceptance.

Evidence-continuous boundary control couples this graph to enforcement. Before a protected transition takes effect, the responsible enforcement point checks the current request against policy and checks those predecessor properties that are necessary for that transition. It then emits evidence of the decision and disposition. Authorization is therefore neither inherited solely from a prior allow decision nor replaced by a provenance record. The central hypothesis concerns the incremental value of these cross-boundary dependencies, not the independent value of authentication, encryption, versioning, or documentation.

### 4.2 Conceptual evidence contract

For a scoring episode S, the required evidence connects the identity and delegation, policy decision, source data versions, feature definition and value snapshot, model artifact and release approval, scoring event, workflow presentation, and human disposition. Several reads or features may contribute to one score, and one score may have several presentations. The structure is therefore a graph rather than necessarily a linear sequence. Each node carries a unique event or artifact identifier within an issuer namespace, event time, recording time, artifact and version references, and typed predecessor links. A case-level correlation identifier joins records but cannot replace node identities or establish causal order.

The decision record binds the subject and workload, action, purpose, patient/encounter scope where relevant, destination, policy version, material attributes, consent reference when applicable, decision, reason, obligations, and expiry or reevaluation conditions. A model transition additionally identifies feature compatibility, model digest, release approval, intended-use configuration, and deployment target. The scoring record identifies the input cutoff and output; the presentation record identifies what context was actually displayed, where, and to which authorized session. A human-action record refers to that presentation, not merely to the patient's chart. Only proportionate, lawful action metadata should be retained.

An expected-event manifest or state model is required independently of the emitted logs. It specifies which events and links should exist for each permitted, denied, failed, or abandoned path. Reconciliation can then detect missing acknowledgments, duplicated events, wrong predecessors, or unauthorized alternative paths. Measuring completeness against the set of records that happened to arrive would make the theory self-confirming. Asynchronous processing requires explicit pending states and timeout rules; ordering must use causal identifiers and clock-uncertainty bounds rather than assuming perfectly synchronized timestamps.

An illustrative transition begins with a request and proceeds to denial, an indeterminate decision, or authorization. An authorized request is followed by recorded execution success or failure, with the corresponding evidence marked as confirmed or pending. A timeout remains unresolved rather than becoming a successful completion. Branch-specific expectations are specified independently: a denied read does not require a downstream score, and displaying an advisory score does not establish that a clinician acted on it. Evidence continuity is assessed at a declared observation cutoff, with legitimate pending states, broken prerequisites, and missing capture reported separately. The expected graph cannot be redefined after observation merely to match the retained records.

### 4.3 Trustworthiness and residual limits

A trustworthy chain requires authenticated producers, appropriately protected keys, version-addressable artifacts, integrity protection over defined bytes or canonical representations, access-controlled evidence storage, and retention sufficient for authorized reconstruction. Append-only storage, independently administered collectors, signed checkpoints, and durable transfer acknowledgments are possible mechanisms, not universally required products. A digest detects change only relative to a trusted reference; a signature attributes a signed statement to a key, not to an infallible observer. A compromised producer can sign false evidence. Loss before capture may remain invisible without an independent expected-event record or observation.

Distributed execution creates an additional limitation: an action and its remote audit record are not automatically atomic. A durable intent/outbox and acknowledgment reconciliation can reduce the gap, but this is an implementation option to be tested. The architecture requires the access decision before a protected effect, and explicit handling of incomplete evidence afterward. An optional AI result may be withheld when critical prerequisites cannot be established; failure of the evidence service must not silently authorize protected access or unnecessarily disable ordinary clinical care. Degraded-mode policy and recovery ownership are part of the contract.

The proposed mechanism is reduction of hidden cross-boundary inconsistency. It is distinct from, but dependent on, the quality of local controls. It cannot establish source-data truth, clinical validity, legal adequacy, or appropriate clinician behavior. More linkage also creates sensitive metadata, additional dependencies, and possible bottlenecks. A finding that these costs outweigh reconstruction or control benefits would count against the proposed operating model.

## 5 Eight requirements and a justified five-layer decomposition

### 5.1 Architectural requirements

Table 2 consolidates the requirements, control responsibilities, and expected evidence. R1 concerns versioned interoperability; R2 human and workload identity; R3 purpose- and policy-aware authorization, including consent where applicable; R4 operational and analytic separation; R5 protection of data and AI artifacts; R6 the governed model lifecycle; R7 cross-domain monitoring; and R8 incremental hybrid deployment. These are author-derived requirements for the proposed architecture, not an exact set prescribed by a standards organization. Supplementary Table S2 provides the detailed source-level reasoning and residual limitations.

**Table 2:** Consolidated requirements, primary responsibility, and observable evidence. The eight requirements and their grouping are author-derived; source-level reasoning and residual limitations are in Supplementary Table S2.

Requirement	Primary layers and control responsibility	Evidence expected if implemented
R1: Standards-based interoperability	L1/L2; versioned interfaces, profiles, terminology, bounded retrieval and rejects (1, 34, 43)	Request/delivery manifest; source versions; profile/terminology package versions; conformance and reject records.
R2: Policy-driven identity	L3 with distributed enforcement; unique humans/workloads, bounded delegation and scopes (5, 6)	Identity and delegation reference; decision ID; scope and expiry; attributable execution or denial.
R3: Purpose- and policy-aware authorization	L3 and L4/return gates; lawful/governance basis, purpose, obligations and consent where applicable (11, 39, 40)	Versioned basis and policy; applicable consent reference or reason not applicable; decision/obligations; exceptions.
R4: Operational/analytic separation	L4 with L3; controlled transfer and separate administrative responsibilities (6, 35)	Asset and access inventory; transfer manifest; permitted path and downstream scope; recovery evidence.
R5: Protected AI data operations	All layers; protect identities, data, artifacts, keys, and evidence; minimize unnecessary copying (12, 23, 35)	Integrity references; key/producer identity; artifact inventory; protected evidence and retention records.
R6: Governed model lifecycle	L4 execution and L5 approval; versioned features, intended use, releases, rollback and retirement (7, 28, 30)	Feature/model versions; evaluation references; approval/configuration; score-to-release link; rollback record.
R7: Cross-domain monitoring	L5 with all producers; reconcile security, data, model, workflow and response evidence (7, 36, 37)	Expected/observed event reconciliation; alerts; adjudicated incident/action records; monitoring ownership.
R8: Incremental hybrid deployment	L1-L5; bounded connectors and staged introduction without replacing the EHR (6, 16, 52)	Environment/use-case scope; readiness decisions; fallback and recovery plans; expansion reassessment.

### 5.2 Why these five responsibilities, not five mandatory products

The decomposition follows differences in authority and change cadence. R1 and R8 require source stewardship and a stable exchange boundary; these motivate L1 and L2. R2 and R3 require runtime identity, policy decisions, and enforcement coordination; these motivate L3. R4-R6 require governed storage, transformations, and model execution; these motivate L4. R6 and R7 require accountable interpretation of evidence, approval, monitoring, and escalation; these motivate L5. R5 spans all layers because protecting evidence and artifacts cannot be delegated to a single storage component. R8 constrains how all layers are introduced without replacing the EHR.

This is one defensible responsibility decomposition, not a proof that five layers are uniquely necessary or universally sufficient. It separates clinical stewardship, representation, request authorization, execution, and governance so that omissions and shared-administrator bypasses can be identified. Smaller implementations may combine components and larger ones may subdivide them without changing the contract. Complete requirements coverage cannot establish clinical or security sufficiency. Applications are external consuming workflows with controlled interfaces, not an unnumbered sixth layer; Figure 1 depicts that distinction.

**Figure 1:**
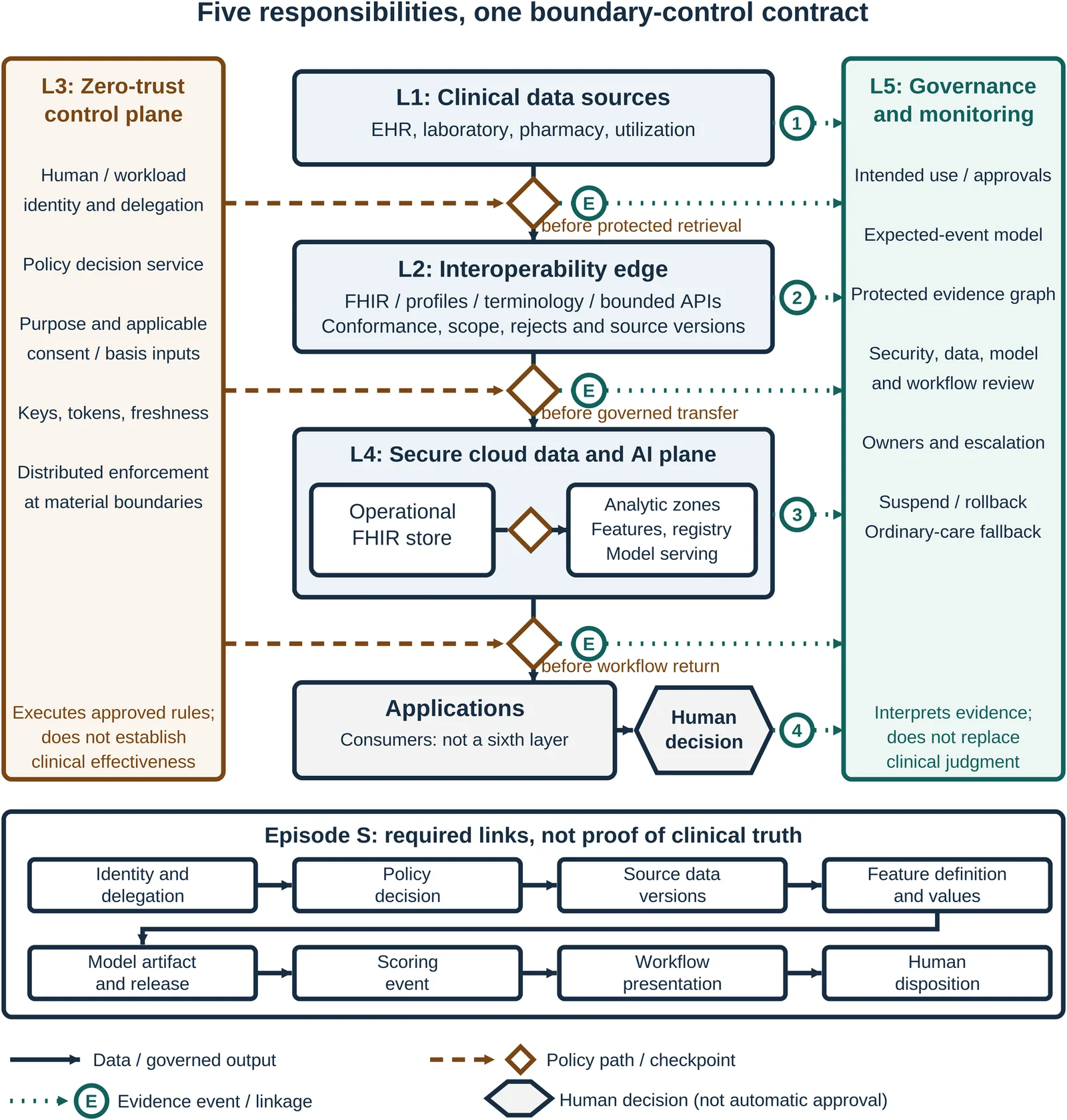
Five-layer reference architecture and the proposed evidence-continuous boundary-control contract. L1 supplies authoritative clinical sources; L2 mediates versioned exchange; L3 supplies policy decisions to distributed enforcement points; L4 separates operational FHIR storage from analytic and model services; and L5 governs and reconciles evidence across the pathway. Applications are external consumers, not a sixth layer. Solid arrows indicate data or governed outputs; dashed paths and diamonds indicate policy coordination and checkpoints; dotted paths and circles indicate evidence production/linkage; the hexagon marks human decision-making. Policy checks precede protected retrieval and later material transitions. Numbered circles group illustrative producer events rather than enumerate every required log. The lower panel shows the episode-level dependency chain. The figure is a conceptual responsibility model, not a deployed network or an assurance certificate.

### 5.3 L1 and L2: sources and interoperability edge

L1 contains authoritative clinical sources, including the EHR, laboratory, imaging, pharmacy, and relevant utilization systems. Its responsibilities are source ownership, authorized extraction scope, identity matching, temporal meaning, and change notification. The proposal does not presume that an authoritative system contains complete or correct data. Source uncertainty and availability limitations must remain visible downstream. EHR data-quality literature distinguishes several task-dependent dimensions rather than equating syntactic acceptance with fitness for reuse (34).

L2 provides the exchange boundary: FHIR APIs, selected profiles, terminology packages, SMART entry points, bounded export or connector interfaces, request limits, reject handling, and technical conformance checking. Its distributed policy enforcement point calls L3 before protected retrieval or prefetch. This layer is an interface responsibility; the operational FHIR store in L4 is a storage/execution responsibility, not a second competing interface. L2 records the requested and delivered scope, source version or snapshot locator, selected profile and terminology versions, and rejects. Conformance checking is verification against a technical specification, not clinical validation.

### 5.4 L3: zero-trust control plane

L3 supplies human and workload identity, scoped credentials, policy decision services, key/token services, and the context required by enforcement points at L1/L2, L4, and application return. Enforcement is distributed even when policy administration is centralized. A current token proves neither a continuing care relationship nor an approved downstream purpose. Reassessment is required when a material attribute changes, at configured expiry, and at downstream transitions identified in the use-case policy. A cached decision requires an explicit scope and freshness rule; calling the architecture zero trust does not eliminate stale-state risk.

The control plane evaluates approved rules; it does not decide that a model is clinically effective, interpret every jurisdiction's law, or determine the acceptable trade-off between denial and treatment delay. Those decisions belong to organizational governance and qualified local decision-makers. L3 can enforce a release approval from L5, but it cannot manufacture that approval. This is the boundary between zero-trust mechanisms and healthcare AI governance.

### 5.5 L4: secure cloud data and AI plane

L4 separates the operational FHIR store from analytic zones with distinct identities, transfer routes, access, retention, and change controls. Feature development, model development, and clinical serving may require further separation. The separation is logical and administrative; network/account separation is selected where local risk warrants it. Shared privileged access can defeat an otherwise attractive network diagram and must be modeled explicitly.

Within L4, feature definitions include code/terminology versions, input windows, missingness handling, and ownership. Registry records identify model digests, dependencies, intended use, evaluation artifacts, approval state, deployment configuration, rollback, and retirement. Transfer, feature, release, and scoring enforcement points check applicable policy and linked prerequisites, emitting separate decision and execution records. Encryption, secret management, artifact integrity, restricted egress, and recovery controls protect the operations; applicable health-data safeguards remain organizational obligations rather than properties conferred by cloud hosting (35). No specific vendor, lakehouse, service mesh, or feature-store product is required.

### 5.6 L5 and consuming applications

L5 owns governance interpretation and monitoring across security, data, model, and workflow signals. It connects alerts to named owners, review thresholds, patient-safety escalation, suspension, rollback, or policy revision. Drift is a signal for investigation, not proof of harm; absence of a signal is not proof of safety. Empirical calibration-drift findings in other tasks motivate continued measurement, not claims about this readmission example (36).

Applications receive an authorized, context-bound output and may record a human disposition. They do not inherit broad access to raw audit stores. Clinical judgment remains outside the model's authority; recording disagreement does not authorize software to override that judgment. L5 maintains the distinction between a technical failure, an unresolved governance question, and legitimate clinical disagreement, consistent with a sociotechnical view of health information systems (37).

## 6 Asset-centered threat model and qualitative prioritization

### 6.1 Conditional scope and rating method

The threat model concerns a hypothetical hospital-connected cloud pathway with human and workload identities, an operational FHIR service, a separate analytic environment, a model registry, a scoring endpoint, and a clinician-facing return path. Actors and failure sources include external attackers, compromised insiders or applications, privileged administrators, supply-chain compromise, misconfiguration, and routine operational failure. No hospital inventory, incident frequency, attacker assessment, or measured control effectiveness was available. The priorities in Table 3 are therefore conditional planning judgments, not a hospital risk assessment.

**Table 3:** Conditional qualitative threat priorities for the hypothetical pathway. H, high; M, moderate; L, low. Priority A/B/C and uncertainty are defined in Section 6. Ratings are planning judgments, not measured probabilities or a real hospital risk assessment.

Path/priority	Conditional likelihood, impact, uncertainty	Control and remaining failure mechanism
T1 Identity or purpose misuse/A	Likelihood H: exposed requests and changed context. Impact H. Uncertainty H: local identity/policy quality unknown.	Bound identity/purpose/scope across gates. Valid-session misuse, stale relationships, and incorrect approved policy can remain.
T2 Operational/analytic outage or propagation/A	Likelihood H: shared dependencies and ordinary failures. Impact H. Uncertainty H: topology and recovery unknown.	Separate planes, bounded transfer and ordinary-care fallback. Shared administrators or unavailable identity/evidence services may defeat isolation or delay work.
T3 Semantic or feature mismatch/B	Likelihood H: source/profile/configuration changes. Impact M in this bounded advisory use; H if care dependence grows. Uncertainty H.	Version manifests and compatibility checks. Syntactically valid but clinically wrong inputs remain possible; elevated impact would raise priority.
T4 Model/release or supply-chain compromise/B	Likelihood M: build/registry privileges or additional prerequisites. Impact H. Uncertainty H.	Digests, approved releases, independent deployment checks. Compromised producers or unsafe approved models can pass technical checks.
T5 Wrong-context or altered presentation/B	Likelihood M: interface/context failures. Impact H. Uncertainty H.	Return gate, encounter binding, content evidence, human review. An intact but misleading result can still induce unsafe reliance.
T6 Evidence loss, tampering or alert overload/A	Likelihood M: capture/collector/administrative failure. Impact H across dependent controls. Uncertainty H.	Independent expectations, protected storage, reconciliation and response owners. Pre-capture loss, forged producer evidence and privacy concentration remain.
T7 External device/IoMT input extension/C	Likelihood L in base case: interface not assumed. Impact may be H if enabled. Uncertainty H.	Assess device identity, quality and new trust boundary before adding it. This conditional extension cannot be treated as already covered.

NIST risk guidance motivates consideration of threats, vulnerabilities, likelihood, impact, and uncertainty (38). Because no local inventory or incident-frequency data were collected, the qualitative likelihood labels here are explicitly conditional exposure/feasibility proxies, not estimates of occurrence probability. High denotes a repeatedly exposed route or ordinary workflow error with a plausible single failure; moderate requires privileged access or additional conditions; low requires an interface not assumed in the base case. High impact includes disrupted care, extensive protected-data exposure, or materially misleading clinical information; moderate impact denotes bounded disruption or limited exposure; low denotes readily reversible effects without protected-data or clinical consequences.

Priority A denotes high impact with high conditional likelihood, or a common dependency capable of invalidating multiple controls. Priority B denotes high impact with additional prerequisites, or moderate impact with a readily exposed path. Priority C denotes a conditional extension outside the base use case. Uncertainty is reported separately: high means local exposure or control effectiveness is unknown; moderate means the mechanism is well specified but context remains unavailable. No ordinal multiplication or numerical risk score is used. A local assessment could legitimately reverse the ordering.

### 6.2 Prioritized paths and residual reasoning

Table 3 gives highest priority to identity and purpose misuse, operational availability, and failures of shared evidence dependencies. Semantic corruption, unsafe model release, and altered workflow presentation are also considered because their integrity consequences may affect care even without an attacker. Supplementary Table S3 maps assets, actors, exposed interfaces, controls, independent evidence, and residual failures. It includes shadow analytic copies, registry and build compromise, evidence tampering, and shared administrative bypasses. Prompt injection is outside this non-generative readmission example.

The proposed controls leave identifiable failure mechanisms. Short-lived credentials do not prevent valid-session misuse; segmentation does not stop an authorized export or a shared administrator; and a valid signature does not establish model suitability. Evidence may be lost before capture, while centralized records may expose sensitive metadata. Authorization-service failure can delay legitimate work, but automatic fail-open behavior can defeat purpose limitation. Evaluation must therefore include dependency outages and false denials alongside malicious scenarios, while preserving ordinary clinical assessment when the AI service is unavailable.

No post-control numerical risk reduction is claimed. A future local risk owner must reassess T1-T7 against actual identities, topology, data sensitivity, clinical dependence, and recovery tests. Enabling device inputs, widening patient scope, changing a release, losing evidence capture, or increasing reliance on the score triggers reassessment. Supplementary Table S3 assigns proposed security, data, model, and clinical responsibilities. A listed control does not, by itself, justify lowering a priority.

## 7 Purpose- and policy-aware authorization, including consent where applicable

The term authorization has two distinct uses. Technical authorization is a system decision permitting an action; a legal authorization or consent is a particular basis or instrument recognized in the applicable setting. A FHIR Consent representation may contribute to a decision but does not determine all lawful uses, interpret law, or implement enforcement by itself (11). The policy service must combine the relevant legal/governance basis, organizational policy, purpose, identity, patient relationship, data category, destination, time, and consent restrictions where applicable.

US HIPAA guidance distinguishes voluntary consent for treatment, payment, and healthcare operations from an authorization required for certain uses not otherwise permitted (39). Research may require an appropriate authorization or a qualifying waiver or other permitted pathway; research-participation consent and permission to use protected health information are not interchangeable (40). This illustration does not classify every model-development activity as operations or research. That classification, any additional federal/state requirements, and institutional approvals must be resolved locally before the technical policy is configured. An organization's preference alone cannot create a lawful basis.

Data minimization is an architectural objective throughout the pathway. It is not described as a universal HIPAA minimum-necessary requirement: that legal standard has exceptions, including certain provider treatment disclosures and requests (41). Likewise, pseudonymization or tokenization does not automatically establish de-identification (42). The evidence record should identify the determined privacy status and applicable restrictions rather than asserting that removing an identifier makes unrestricted reuse permissible.

At each material use, the policy decision records the applicable basis and its versioned evidence. Where no consent is required for that permitted use, the record explicitly states that consent was not the operative basis; it must not fabricate a Consent resource. A withdrawal or change triggers reevaluation of affected future uses within the legally and operationally defined scope. It does not imply automatic deletion of legally retained clinical records, reversal of completed processing, or erasure of influence from a previously trained model. Those are separate retention, research, and model-governance questions.

Emergency or break-glass access requires a separate, legally permitted and organizationally approved path with bounded scope/duration, justification, attributable identity, and retrospective review. It is not a generic bypass of all controls, and a readmission-risk service should not invoke it simply to keep optional scoring available. An indeterminate policy state is distinguished from an explicit denial and routed to the defined safe fallback or authorized resolution process; it is never silently treated as permission.

## 8 Concrete FHIR and non-FHIR evidence linkage

### 8.1 Release and profile context

The worked linkage is pinned to FHIR R4 version 4.0.1. Where applicable clinical resource profiles are needed, the reference context is US Core 9.0.0, an R4 implementation guide (43). This selection is a reproducible illustration, not a claim that every EHR supports that guide. Consent, AuditEvent, RiskAssessment, and external AI-artifact links use the base R4 definitions with the local conceptual constraints described below. No new implementation guide, validated profile package, or executed clinical-scoring instance is supplied for this linkage; the companion exchange artifact has the separate scope defined in Section 2.4. SMART 2.2.0, Bulk Data 3.0.0, and CDS Hooks 2.0.1 are separately pinned interface references, not evidence that those resources are cross-version equivalent (2–4).

### 8.2 Symbolic scoring episode and linkage contract

All identifiers in Table 4 are symbolic; no patient record, numerical risk score, executed decision, or real approval is represented. Episode S links distinct policy decisions PD-read, PD-feature, PD-score, and PD-return. Each decision refers to the specific request and applicable policy version; a relevant Consent version C is referenced only when it contributes to that decision. Source manifest D identifies the exact retrieved versions, query scope, input cutoff, profile/terminology package versions, and any unavailable or rejected inputs. Feature artifact F identifies both the definition and the values used for S. Registry record M and release A identify the approved model artifact and configuration.

**Table 4:** Symbolic R4 evidence linkage for scoring episode S. These are design identifiers and field-level conventions, not generated patient data, executed decisions, validated instances, or claims of implementation. Detailed preservation rules appear in Supplementary Table S4.

Artifact/identifier	FHIR R4 representation where applicable	Required linkage/non-FHIR record
Identity I; decisions PD-^*^	Device/Practitioner references where appropriate; applicable Consent version C (11)	External decision binds actor/delegation, purpose, action, patient/encounter scope, policy/basis versions, obligations and transition S. Consent is not always applicable.
Source manifest D	Version-specific source references, including server namespace; otherwise retained acquisition snapshot (45)	Exact delivered scope, input cutoff, profile/terminology versions, rejects and acquisition integrity reference.
Feature F; model M; release A	DocumentReference for controlled external feature/registry/approval artifacts (44)	Definition and value snapshot; model digest and dependencies; actual approval/configuration reference if implemented, not an invented FHIR Model resource.
Score S	RiskAssessment.identifier; subject; encounter; occurrenceDateTime; prediction; basis; performer Device (14)	Basis refers to source/feature artifacts; scoring record links PD-score, D, F, M, A and output. No numerical score is supplied here.
Provenance of S	Provenance.target = versioned RiskAssessment; entity.what = input/manifest references; agent; occurred[x]; recorded (12)	The source role is used for contributing entities. policy identifies a policy/plan URI, not a substitute for an event-specific decision record.
Access/decision/return events	AuditEvent.id, agent, source.observer, entity.what; entity.detail.type/valueString for local correlation/decision keys (13)	Links S and relevant PD-*. Technical outcome is separate from policy allow/deny. No generic AuditEvent.identifier is assumed.
Presentation U; action H	External workflow log; optional locally supported Task referencing the clinical work.	U binds S, patient/encounter, authorized session and displayed context/version. H refers to U. Missing action does not imply rejection; display does not imply comprehension.
Integrity envelope	DocumentReference points to a signed external manifest; base R4 Attachment.hash is not repurposed (46)	Issuer/key, digest algorithm, canonicalization, predecessor IDs, event/record times and retention policy. Integrity does not prove clinical correctness.

* denotes the set of transition-specific policy-decision identifiers associated with episode S, including PD-read, PD-feature, PD-score, and PD-return.

The RiskAssessment for S has a business identifier, subject and encounter, scoring occurrence time, outcome horizon, and appropriate status. Its basis links the relevant source resources or a DocumentReference for the feature manifest; its performer can identify the scoring Device (14). A Provenance record targets the version-specific RiskAssessment and links source and external artifact references through its entity records, with responsible agents and activity/recording times (12). DocumentReference can describe non-FHIR artifacts and their controlled locations (44). It is a reference mechanism, not a model-registry standard or evidence that an approval occurred.

AuditEvent records capture access, decision, scoring, and return activity. R4 does not provide a generic AuditEvent.identifier element: the resource ID identifies the event record, while entity references and locally specified entity.detail entries carry S and the applicable decision ID (13). These local detail names are conventions of this conceptual contract, not new standard elements. Technical execution outcome and policy allow/deny are distinct: a successfully processed request can correctly result in denial. The independent expected-event manifest records which deny, failure, or successor records should then exist.

Presentation U links S to the authorized patient/encounter context, destination, displayed model/release information, interface-template version, event time, and a protected snapshot or digest of displayed content. Human disposition H links to U and records an action such as acceptance, disagreement, deferral, or a documented workflow step when collection is proportionate. A display event is not proof that a clinician read or understood the result; no response is not a rejection. Task may represent an assigned workflow action where locally supported, but neither a CDS card nor Task is required to store every audit detail.

### 8.3 Integrity, retention, and translation constraints

A source reference must include its server namespace and version or resolve to an immutable retained snapshot. FHIR history operations do not guarantee that every server retains all historical versions indefinitely (45). When a source cannot provide a stable versioned read, the acquisition manifest must bind the exact received representation, acquisition time, and integrity reference, with its weaker provenance made explicit. Raw bearer tokens and unnecessary clinical payloads should not be copied into logs.

The contract can use signed external manifests with a stated modern digest algorithm and canonicalization rule. It must not place a SHA-256 value into R4 Attachment.hash and label it standard-conformant: that field is defined for SHA-1 and is not a digital-signature assurance mechanism (46). The stronger manifest digest remains in the explicitly described external artifact, linked through DocumentReference. Hash equality establishes consistency of the chosen representation, not the truth of a clinical observation.

Table 5 summarizes consequential R4/R5 differences; Supplementary Table S4 gives the mapping conditions. Consent changes affect subjects, dates, statuses, policy representation, and permit/deny structure (11, 47). Provenance changes include optional recording time in R5 and altered authorization/role representation (12, 48). AuditEvent reorganizes event coding, occurrence and outcome fields (13, 49), while RiskAssessment changes reason representation and allowable performer references (14, 50). These are not resolved by changing a version label.

**Table 5:** Consequential R4-to-R5 mapping issues for the worked evidence chain. This is a bounded semantic crosswalk, not a complete conversion specification or a claim of compatibility.

Resource	Relevant difference	Condition for preserving continuity
Consent (11, 47)	R4 patient/scope/dateTime and policy/provision representation differ from R5 subject/date, regulatoryBasis/policyBasis, decision and provision structure; statuses also differ.	Do not rename fields mechanically. Preserve restrictive effect, scope, basis, purpose and time semantics. R5 execution date is not a truncated R4 creation timestamp. Unsupported rules remain unresolved.
Provenance (12, 48)	R5 makes recorded optional; reason becomes authorization; entity roles change. Device/RelatedPerson are removed from agent.onBehalfOf targets.	Keep the contract's recording time and version links. Preserve delegation in a governed typed attestation; do not falsely substitute a permitted target type. Reject unresolved semantic loss.
AuditEvent (13, 49)	R4 type/subtype, period, outcome and purposeOfEvent are reorganized into R5 category/code, occurred[x], structured outcome and authorization.	Preserve event meaning, outcome coding, issuer/actor and local decision/correlation keys. Successful event processing must not be recoded as a policy allow.
RiskAssessment (14, 50)	R4 reasonCode/reasonReference become R5 reason (CodeableReference); performer reference targets broaden.	Preserve identifier, subject/encounter, horizon, occurrence, basis, output interpretation and scorer/release links. Target-type availability alone does not justify a semantic substitution.
All translated artifacts	Translation/reserialization changes representations and may invalidate source-byte hashes/signatures.	Retain original bytes and verification context; issue a new mapping attestation with source/target/profile/terminology versions. Test policy/semantic fixtures and reject unresolved loss.

A translation must preserve the original artifact and its integrity evidence, record the mapping/profile/terminology version, and issue a new transformation record for the resulting representation. Reserialization normally changes bytes; it cannot inherit the original signature as proof of the translated object. Semantic fixtures must test preservation of scope, time, purpose, restrictive policy effects, identities, and links. Unknown codes or unsupported policy structure require explicit unresolved handling, not a permissive default. Evidence continuity across releases is conditional on this preservation and on retained resolvability; dropping a restriction, losing an identifier namespace, omitting required timestamps, or discarding the source representation can break it.

A further identity-sensitive example concerns Provenance.agent.onBehalfOf: R4 permits Device and RelatedPerson targets that R5 removes from that element (12, 48). A workload delegation must not be recast as a practitioner or organization merely to pass a target schema. Preserve the original reference, role, issuer and delegation chain in a versioned external delegation attestation, while keeping the R5 element conformant; mark unresolved semantic loss and withhold a dependent protected transition. R5 schema validity alone does not preserve the R4 attribution claim. Translation tests must therefore cover actor/delegation loss as well as consent, time, entity-role, and outcome changes.

## 9 Illustrative readmission-risk workflow and human action

### 9.1 Intended setting and decision, not a clinical intervention claim

The illustration concerns an adult general-medicine discharge-planning team. A care-coordination nurse sees a non-interruptive risk summary in the discharge-planning worklist; the responsible hospitalist can review it during discharge planning. Its intended use is to prioritize review of transitional-care needs, such as medication reconciliation or follow-up arrangements under an existing care pathway. The output does not independently determine discharge timing, deny services, place orders, or diagnose a patient. Readmission prediction and the benefit of any resulting intervention are different questions; the historical readmission literature illustrates the importance of matching the model to its use and timing (51). No specific score, threshold, model performance, or intervention effect is assumed.

The scoring cutoff t0 is the time at which that planning review is requested. Only information available by t0 is eligible. Elapsed stay is not replaced with a future final length of stay; planned disposition is not mistaken for eventual disposition; subsequent follow-up and readmission outcomes cannot enter the features. Claims and external utilization are optional only when available and authorized at t0, not silently assumed complete. Intended population, prediction horizon, exclusions, feature windows, and the consequences of missing data belong in the model's approval configuration.

### 9.2 Controlled seven-stage pathway

Figure 2 shows seven stages, with authorization checkpoints before protected effects rather than after extraction. First, the workflow creates S and an authenticated request; L3 evaluates purpose, subject/workload, relationship, scope, destination, policy, and consent where applicable. This applies to protected prefetch as well as an explicit subsequent API call. A denied or indeterminate request records that disposition and uses the approved ordinary-care fallback.

**Figure 2:**
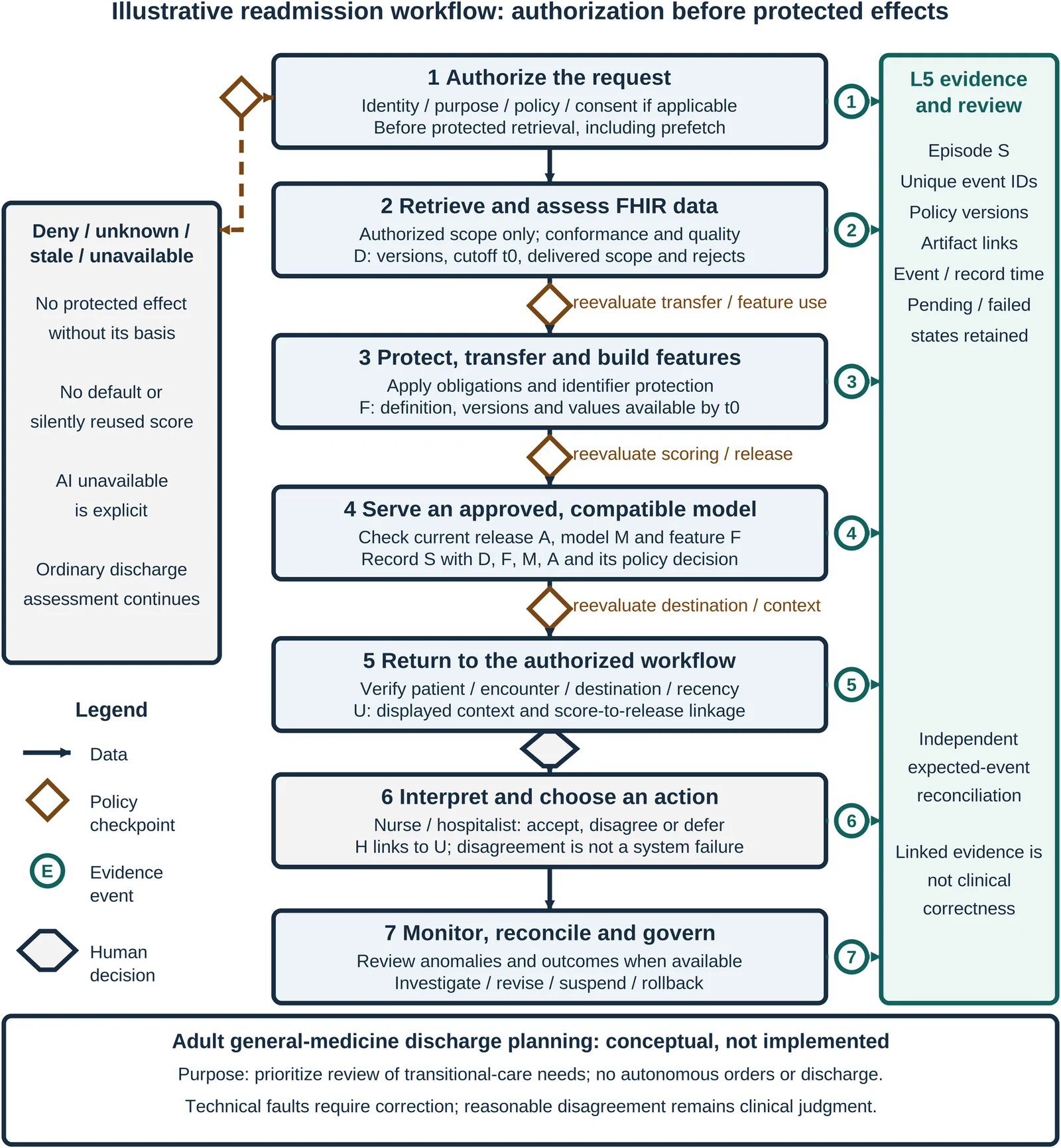
Seven-stage illustrative adult readmission-risk workflow. An authenticated, purpose- and policy-aware request is evaluated before protected retrieval, including prefetch. Retrieved data are assessed and versioned; transfer/feature, scoring/release, and destination/context checkpoints precede their protected effects. A care-coordination nurse or hospitalist receives the contextualized output and may accept, disagree, defer, or initiate an appropriate workflow action. Numbered circles denote evidence events linked to episode S and independently specified expectations. The symbol legend is common to Figures 1,2; evidence events appear here as numbered circles. The illustrated denial/unknown/unavailable branch represents the fallback rule applicable at every relevant checkpoint: do not perform an unauthorized protected effect or silently substitute a default/stale score; continue ordinary discharge assessment. Technical mismatch and legitimate clinical disagreement have different dispositions. No implemented service, risk estimate, or clinical effectiveness is represented.

Second, only an authorized scope is retrieved through L2. Technical conformance and temporal/data-quality checks produce the acquisition manifest D and reject records. Third, the transfer/feature checkpoint reevaluates applicable scope and purpose before governed transformation or analytic transfer; data protection and minimization obligations are applied, and F records the feature definition and values. Identifier protection is chosen for the approved use; necessary clinical reidentification is itself controlled.

Fourth, the scoring checkpoint confirms the permitted use, feature compatibility, and current release A of model M. The server does not infer authorization from a model's presence in a registry. The resulting scoring event links S, the applicable decision, D, F, M, and A. Fifth, the return checkpoint verifies the authorized destination, patient/encounter match, release/context freshness, and required evidence before presentation U. A local discharge-planning trigger or SMART interface may implement this interaction; no universally supported discharge-specific CDS hook is presumed.

Sixth, the nurse or hospitalist interprets the result with the clinical record and may accept, disagree, defer, or initiate a permitted workflow action. H refers to U where recorded. Seventh, L5 reconciles evidence and reviews security, data, model, and workflow signals. Monitoring can lead to investigation, interface redesign, policy correction, rollback, suspension, or retirement; it does not automatically label a clinician's disagreement an error.

### 9.3 Unavailability, disagreement, and appropriate reliance

If the model, required input, authorization decision, or critical evidence prerequisite is unavailable or stale, the interface says that the AI assessment is unavailable and why at a useful level. It does not show a default low-risk value or silently reuse an old score. The team continues its usual discharge-planning assessment. Optional AI availability must not become a hidden prerequisite for ordinary care.

A wrong-patient association, incompatible feature version, unapproved release, or inconsistent displayed score is a technical/governance failure requiring suppression or correction and an incident path. A clinician who sees the intended result and reasonably chooses a different action is exercising judgment. A compact disagreement reason can be offered without mandatory free-text burden; override rates alone cannot identify error or noncompliance. Disagreement may reveal omitted context, an unsuitable threshold, or a different intervention opportunity and needs adjudication, not automatic retraining labels.

The display should prioritize intended use, input recency, material limitations, and the ability to question the result. Confidence or uncertainty information should appear only when its interpretation is justified for the model and setting; statistical confidence is not a substitute for verified provenance, and provenance is not proof of clinical correctness. Detailed technical evidence should be available by progressive disclosure rather than a reassuring but uninterpretable proof badge. P6 tests both inappropriate following of incorrect advice and inappropriate rejection of useful advice, including the possibility that polished contextual information worsens reliance.

## 10 Six falsifiable propositions

### 10.1 Common comparison and decision rules

The primary comparator M0 is a well-run contemporary platform with distinct workload identities, scoped credentials, current policy enforcement, encryption, segmentation, versioned features/models, approvals, rollback, multichannel monitoring, and a usable clinical interface with an override path. Its actual configuration and existing cross-stage links must be inventoried before comparison. Native safeguards must not be removed to manufacture a gap. M0 and the proposed condition M1 receive matched workloads, source snapshots, policies, model artifacts, clinical cases, compute/latency budgets, staffing, and training. Where M0 already enforces the complete contract, M1 has no incremental contract intervention; an equality finding is substantive counterevidence to added value, not a reason to weaken M0.

Where a documented contract gap exists, M1 adds the missing prerequisite checks and expected-event reconciliation while retaining M0's other safeguards. Mechanistic studies may separately use a join-only condition or remove one link from a common reference implementation, but such ablations belong in synthetic/simulated studies, are explicitly labeled degraded variants, and do not establish superiority over a production platform. Named architectures inform comparator selection, not assumed feature absence. A benefit caused by weaker M0 authentication, omitted monitoring, extra M1 staff, or a different clinical interface is confounded rather than evidence for the contract.

A future protocol must specify the unit of analysis, independent reference standard, materiality rules, sampling frame, primary endpoint, meaningful benefit margin, harm tolerances, observation window, sample-size rationale, and analysis before generating data. Each proposition below has one primary endpoint; secondary outcomes cannot compensate for a failed primary comparison. Support requires an uncertainty interval showing benefit beyond the prespecified margin, with all designated harm bounds satisfied. Practical equivalence, a meaningful reversal, or unacceptable harm provides counterevidence. Wide intervals, unresolved reference labels, no relevant test opportunities, or unmatched conditions are inconclusive; nonsignificance alone does not establish equivalence. Paired or clustered analysis must account for repeated requests, shared incidents, and clinician-level observations. Numerical margins depend on the intended setting and require prospective justification. Each comparison below assumes that the stated difference has been documented; existing comparator safeguards are preserved when no such difference is present.

### 10.2 P1: identity-bound cross-boundary authorization

Mechanism and intervention: M1 binds principal/delegation, requested action, and approved scope to the exact successor request so that an otherwise valid identity cannot silently acquire a different scope across a service boundary. M0 retains the same identity and local access controls but does not verify this predecessor-to-successor binding. A link-removal ablation preserves authentication and token lifetimes.

Independent reference and measurement: designated policy owners approve a separately maintained fixture specifying the permitted actors, purposes, resources, fields, and actions before testing. This fixture, not the system's decision, determines the expected result. The primary prohibited-effect rate is the number of test requests producing at least one fixture-prohibited effect divided by all test requests attempting at least one prohibited effect. This includes an over-broad request that returns forbidden fields alongside permitted content. Over-breadth is any delivered resource, field, or action outside the approved scope. Excess deliveries are also reported per completed request. Attribution accuracy is correctly attributed events divided by all independently observed events, including denials. False-denial rate is reference-permitted requests incorrectly denied divided by all reference-permitted requests. An external harness observes actual effects; gateway and identity logs are supporting evidence, not the reference standard.

Primary endpoint and decision: the prohibited-effect rate defined above is P1's primary endpoint; lower is better. Accurate attribution and reduced excess scope are secondary and cannot substitute for a failed primary contrast. Support additionally requires false-denial and latency bounds to be met. Precise equivalence/reversal, wrongly attributed effects, or exceeded harm bounds count against P1; ambiguous policies or no prohibited-effect test opportunities are inconclusive. Reliable identity resolution and an independently specified scope policy are conditions. Added authentication burden, metadata exposure, failed requests, and workflow delays are measured adverse effects, not reasons to discard cases.

### 10.3 P2: purpose/policy continuity across reuse

Mechanism and intervention: M1 checks the scope, freshness, purpose, applicable consent/authorization basis, and obligations inherited through source, feature, scoring, and return transitions, reevaluating changed attributes. M0 checks current policies locally but does not reconcile the downstream artifact with the basis and obligations of its predecessor. An ablation removes the policy-version/purpose link while retaining the policy engines.

Independent reference and measurement: an independently specified policy state machine supplies permitted and prohibited uses over time, including treatment, approved research, withdrawals, expiries, lawful retention, and separately authorized exceptions. Reference-discordant completed uses are divided by all attempted uses labeled prohibited. False denials use all permitted attempts as denominator. Change-to-effective-enforcement delay is measured from a reference policy change to the first sustained concordant behavior, with never-enforced changes censored and reported separately. External observers and immutable fixture versions provide the reference; decision records and transform/serving logs provide implementation evidence.

Primary endpoint and decision: completed prohibited-use rate is primary for P2. Enforcement delay is a secondary freshness measure, with never-enforced changes reported rather than omitted. Support requires a lower primary rate beyond the prespecified margin without excessive false denial or care delay; a faster update alone cannot rescue unchanged prohibited reuse. Precise nonbenefit, blocked lawful uses, or unacceptable availability burden count against P2. Unresolved legal classification or absent change/transition opportunities is inconclusive. Locally adjudicated legal meaning and update signals are conditions; stale attributes, excessive consent metadata, exception misuse, and inability to reverse completed processing remain limits.

### 10.4 P3: segmentation with evidence-bound transfers

Mechanism and intervention: both conditions separate operational and analytic environments. M1 additionally binds transfer manifests and permitted downstream assets to the authorized transition, checks discrepancies, and supplies linked evidence to containment procedures. The ablation removes transfer linkage while preserving network and administrative segmentation. This tests incremental transfer accountability, not the already established rationale for avoiding a broadly shared environment.

Independent reference and measurement: a test controller fixes the asset inventory, permitted reachability, seeded credentials, attack paths, and compromise start times independently of the defense logs. Unexpectedly reachable assets are counted against all enumerated protected assets probed; unauthorized successful transfers use all prohibited transfer attempts as denominator. Time to containment runs from controller-observed compromise to independently observed cessation of the specified path; uncontained trials are not dropped. Transfer records, network/service probes, egress observations, and incident decisions provide evidence.

Primary endpoint and decision: successful prohibited transfers divided by all prohibited-transfer attempts is primary for P3; reachability and containment time are secondary. A lower transfer rate supports P3 only within recovery, availability, and latency bounds. Precise nonbenefit under matched segmentation, unmodeled administrative bypasses, or unacceptable outages count against the claim. An incomplete controller inventory or unequal attack privileges is inconclusive. Assumptions about shared administrators must be declared and challenged in a separate scenario, not silently excluded. Extra connectors, recovery complexity, staffing cost, and common policy/evidence-service failure are adverse effects.

### 10.5 P4: reconstructable scoring and release integrity

Mechanism and intervention: both conditions version features and models and enforce approved deployment. M1 also binds each score to the exact input snapshot, feature definition/values, model digest, approval, threshold configuration, and presentation. M0 retains local registry and pipeline histories without the episode-level compatibility check. An ablation removes source-to-feature or release-to-score links separately.

Independent reference and measurement: a controller independently records the episode's identity and policy references, source and feature snapshots, digests, approvals, configuration, original output, presentation, and disposition before mismatch injection or reconstruction. This reference manifest is withheld from reconstruction operators. An episode passes reconstruction only when every prespecified required identity, policy, source, feature, model and release, score, presentation, and disposition reference matches the controller-held record within a fixed investigation budget. Correctly recorded pending or not-applicable states are judged against the independently specified branch, not treated as missing clinical actions. The primary rate is passing episodes divided by all sampled episodes. Missing critical links, incorrect joins, failed reconstruction, and lawful unavailability remain nonpassing in this feasibility denominator. A resolvable URI or an internally consistent but incorrect chain does not pass. Replay is assessed separately: exact equality is required for a specified deterministic environment, or a justified preregistered tolerance and relevant decision category for nondeterministic execution. Prohibited release or use is assessed across all reference-prohibited attempts.

Primary endpoint and decision: P4 requires an increase in the correct-reconstruction rate beyond the prespecified benefit margin, with the uncertainty and harm rules in Section 10.1 satisfied. Replay agreement and blocked prohibited releases are secondary. Incorrect reconstruction despite intact-looking links, practical equivalence, or excessive storage, privacy, or serving burden provides counterevidence. Unknown reference truth or unspecified nondeterminism makes the affected replay comparison inconclusive; unavailable historical artifacts remain in the reconstruction denominator. Reference and evidence stores must be separated so that reconstruction cannot obtain the answer directly from the reference manifest. Reproducing a score does not establish its clinical correctness.

### 10.6 P5: cross-domain incident reconstruction and response

Mechanism and intervention: M0 has functioning security, data-quality, model, and workflow monitoring with equivalent analysts and thresholds. M1 adds episode-level linking and expected-event reconciliation to associate incidents across those domains. A correlation-only or withheld-domain-link ablation tests whether the benefit requires the complete contract rather than a better dashboard.

Independent reference and measurement: a controller seeds incidents using a concealed schedule and a fixed set of required facts. Primary detection delay is the mean elapsed time to independently adjudicated detection, capped at a common observation horizon, tau, specified in advance. Every seeded incident is included; an undetected incident is assigned tau. Detection sensitivity is detected incidents divided by all seeded incidents. Incidents that end before tau are retained. False alerts are independently adjudicated nonincident alerts per monitored hour and per episode. Action delay and the proportion of controller-required facts correctly reconstructed are secondary. Unexpected observation loss requires a disclosed sensitivity analysis or an inconclusive comparison, rather than being treated as successful censoring.

Primary endpoint and decision: shorter restricted detection time supports P5 only with incident-coverage, false-alert, and analyst-workload bounds satisfied. Faster triage or improved fact reconstruction alone cannot rescue a failed primary contrast. Precise nonbenefit, erroneous joins, or diversion of response from other incidents counts against P5. Missing controller truth, unequal training/severity, or insufficient common follow-up is inconclusive. Clock alignment, declared observation horizons, and available response capacity are conditions. Concentrated sensitive evidence, staff burden, and false attribution remain adverse outcomes.

### 10.7 P6: contextualized return and appropriate reliance

Mechanism and intervention: M0 already displays a score, intended use, timestamp, ordinary model information, and a human override path. M1 adds concise episode-linked context and actionable warnings about relevant stale, incomplete, or incompatible evidence, with progressive disclosure. The same model outputs and interface quality are used. An ablation supplies generic, equally polished contextual information without verified episode linkage; another removes the warnings while retaining the provenance panel.

Independent reference and measurement: before closed human-factors simulation, a clinical panel independent of the interface designers defines acceptable action sets for correct, incorrect, out-of-scope, and unavailable-advice cases, blinded to allocation and without using the model as the oracle. The primary appropriate-action rate is observed decisions within the panel's acceptable set/all independently adjudicated assigned cases, including cases in which the system withholds advice. Results are stratified by advice condition. Unsafe following is inappropriate model-consistent action/all cases with adjudicated inappropriate advice; under-reliance is failure to take any acceptable indicated action/all cases requiring such action. Clinically reasonable disagreement within the acceptable set is not an error. Comprehension and correctly linked observed actions/all observer-recorded actions are secondary. A single eventual readmission is not a truth label for an individual probabilistic score.

Primary endpoint and decision: a higher appropriate-action rate is required for P6, with no unacceptable worsening of unsafe following, under-reliance, interpretation time, or workflow burden. Higher comprehension, confidence, or reported trust cannot rescue a failed behavioral endpoint. Precise nonbenefit or exceeded harm bounds is counterevidence; disputed reference sets, ceiling effects, or inadequate precision is inconclusive. Matched clinical advice and interface polish, representative users, accessible language, and realistic time pressure are conditions. Dangerous or incompatible advice is tested only in closed vignettes, not knowingly delivered to patients. Equal-polish and warning-removal ablations distinguish evidence checking from persuasive presentation.

## 11 Staged evaluation and implementation gates

Stage 1 would use synthetic FHIR fixtures and independent policy/reference manifests to test conformance, authorization order, denials, revocation/expiry behavior, version compatibility, missing evidence, and replay. Stage 2 would add attack-path simulation, fault injection, and recovery/tabletop exercises, including failures of policy, identity, evidence, and cloud dependencies. Stage 3 would use structured expert challenge and human-factors simulations to assess assumptions, semantic/legal interpretation, workflow plausibility, and P6. Expert opinion could revise the design but would not establish operational or clinical effectiveness.

Stage 4 would introduce a bounded pilot only after technical, governance, privacy, and safety prerequisites were met. It would measure real workflow burden, unavailable-score behavior, subgroup effects, calibration when outcome data are available, operational cost, and incident response. DECIDE-AI can guide reporting of early live clinical evaluation; it is not a certification procedure or a replacement for a study design (52). Stage 5 would assess clinical and patient outcomes prospectively with an appropriate comparative design and local approvals. Technical success at earlier stages does not warrant expansion or a claim of clinical benefit.

Implementation would follow the same gates: harden the exchange boundary; establish bounded identities and a governed hybrid landing zone; add segregated feature/model operations and release evidence; then test one constrained workflow before scaling. Each expansion of site, population, model, or interface changes the assumptions and requires reassessment. A limited-resource organization may combine physical services, but cannot infer that outsourcing a component transfers policy or clinical accountability. Feasibility and staffing costs remain empirical questions.

Future protocols must specify suspension conditions before use: unauthorized protected effects; incompatible or unresolvable critical evidence; wrong-patient/encounter presentation; unapproved release; unavailable rollback; or a clinically material performance, equity, or reliance hazard. Thresholds, timing, exception authority, and ordinary-care fallback must be locally justified. No evaluation study of the complete architecture or P1-P6 has been run, and no corresponding gate is reported as passed in this paper. The companion implementation's technical checks do not establish completion of these prospective stages. Supplementary Table S5 separates proposed measurements from observed evidence, of which none is supplied for the architecture.

## 12 Discussion

### 12.1 What would constitute a contribution

The proposed theory is about a dependency relationship: boundary decisions and lifecycle evidence should refer to the same material episode and compatible artifact versions. Its value would lie in preventing or revealing errors that survive otherwise strong individual controls, and in reconstructing those errors without disproportionate burden. The five-layer diagram is an allocation of responsibilities for this mechanism, not its proof. A differently layered architecture that implements the same contract could instantiate the same theory.

There are credible alternatives. Well-maintained local logs may permit adequate reconstruction without runtime linkage; governance procedures may prevent the relevant mismatches; the event rate may be too low to justify the complexity; or the added dependency may degrade availability. A benefit limited to faster retrospective investigation would narrow, not confirm all, of the prevention claims. Similarly, stronger audit completeness without better appropriate clinical action would support a technical property but not P6 or patient benefit. The propositions should be reported separately rather than combined into an unqualified claim that the architecture works.

The comparison also clarifies the boundary of novelty. Existing systems may already implement equivalent joins, gates, or evidence reconciliation. The literature comparison identifies reported responsibilities and unresolved equivalence questions, not definitive feature absence. Future evaluations should document the actual comparator configuration and explain whether the tested difference is the contract, an implementation-quality difference, or additional staffing and infrastructure.

### 12.2 Limitations and transportability

This paper reports no patient dataset, trained or evaluated clinical model, penetration-test study, independent expert assessment, hospital risk assessment, or clinical evaluation of the complete architecture. The bounded companion software in Section 2.4 makes selected exchange controls inspectable but does not instantiate all five layers, the readmission pathway, or the independent comparisons required by P1-P6. Literature synthesis and source-to-design mapping establish conceptual traceability only. Screening was not exhaustive or independently replicated; some publisher material was accessible only through abstracts or metadata. The architecture's causal and comparative claims remain hypotheses bounded by the available reporting.

Local law, institutional governance, source-data quality, EHR capability, terminology, retention rules, model suitability, intervention capacity, and staffing can change both feasibility and risk. R4/R5 continuity depends on explicit semantic mapping and preserved artifacts, not generic release agnosticism. Security and evidence controls cannot eliminate authorized misuse, compromised producers, unsafe approved models, or undocumented human work. Evidence retention can conflict with minimization and increase the impact of a breach. These are structural trade-offs rather than residual concerns that disappear after more logging.

The readmission example is an explanatory device, not an implemented pathway or a recommendation to deploy a readmission model. Future studies must separately establish model performance, calibration, subgroup behavior, usability, appropriate reliance, cost, and clinical value. Negative findings, failed reconstructions, false denials, missing outcomes, and abandoned workflows are essential observations for testing the theory, not inconvenient cases to exclude.

## 13 Conclusion

Evidence-continuous boundary control proposes that material transitions in clinical AI should couple an applicable policy decision with checkable, version-linked evidence of prerequisites and disposition. The eight requirements and five functional layers allocate this responsibility without claiming a unique or universally sufficient architecture. A version-specific FHIR illustration and six propositions define the hypothesized benefits and the conditions under which the theory would fail. Staged empirical evaluation is needed to determine whether the proposed contract improves security, reconstruction, or appropriate clinical reliance beyond contemporary safeguards at an acceptable operational and privacy cost.

## Code availability statement

The synthetic-data-only Consent-Aware FHIR Exchange Prototype is publicly available as version v1.0.0 under the Apache License 2.0 (9). The release is provided for research, technical evaluation, reproducibility of its bounded demonstration, and independent inspection of selected FHIR exchange, authorization, provenance, audit, and integrity mechanisms. It does not implement the complete proposed architecture or constitute clinical validation or empirical support for P1-P6. The version-specific release is https://github.com/phemmyhorke/consent-aware-fhir-reference-implementation/releases/tag/v1.0.0; its evidence-bearing commit is c5c3f663cafa4d26da779f14d261e9f37d444fa4. The canonical attached distribution is Consent-Aware-FHIR-Exchange-Prototype-v1.0.0.tgz, identified in the release record by SHA-256 adba6da55f4ce68b7b8e00d466c1bf0256cbc3ac247779056627b83bf757c19a. The release record distinguishes that archive from GitHub-generated convenience archives.

## Data Availability

The original contributions presented in the study are included in the article/Supplementary Material, further inquiries can be directed to the corresponding author/s.
