# Supplementary material for: Operationalizing clinical artificial intelligence through secure FHIR pipelines: a zero-trust cloud reference architecture for EHR interoperability
Source: Front Digit Health. 2026 Sep 29;8:1953969. doi: 10.3389/fdgth.2026.1953969 (PMC13658595; doi:10.3389/fdgth.2026.1953969)
Supplement: Supplementary file 1 [file Datasheet1.docx]

Supplementary methods and tables

Search and provenance records: 10 September 2026. Final editorial and source checks: 11 September 2026. The stages are documented separately.

Reading key: S = specification; G = guidance; G/L = official legal explanation; E = empirical report; R = review; T = theory/method. CO1-CO8 are author-defined control objectives, not published standard clauses. M0/M1 are future comparison conditions.

# Supplementary Table S1. Search records and verification stages

Part A preserves the 46 query strings recorded in the first revision log on 10 September 2026. This is an inherited revision-stage record, not an independently rerun search in the final editorial check. A complete candidate-level ledger was not maintained during the earlier conceptual development; no original screening flow or rejected-hit count is reconstructed.

The records describe general web search and direct retrieval of official, publisher, and PubMed/PMC pages. They do not establish a native PubMed or subscription-database search, independent dual screening, or exhaustive retrieval. Literal query terms and recorded search-engine restrictions are preserved. Source-selection preferences are not represented as search-engine filters.

## S1A: First-stage revision queries (all dated 10 September 2026)

| **ID** | **Exact query (all dated 10 September 2026)** | **Purpose** |
| --- | --- | --- |
| Q01 | site.frontiersin.org journals digital health for-authors article-types Hypothesis and Theory 12000 | Article type / scope |
| Q02 | site.frontiersin.org guidelines author guidelines manuscript formatting figures tables generative artificial intelligence disclosure | Formatting / disclosure |
| Q03 | "DeepXplainer: An interpretable deep learning based approach for lung cancer detection using explainable artificial intelligence" | Reviewer 2 title verification |
| Q04 | "Explainable AI-driven IoMT fusion" | Reviewer 2 title verification |
| Q05 | "XAI-DR-GAN" | Reviewer 2 title verification |
| Q06 | "SE-MMFusionGAN" | Reviewer 2 title verification |
| Q07 | "DEPLOYR" clinical machine learning deployment | Named architecture |
| Q08 | "FHIR-DHP" Williams 43847 | Named architecture |
| Q09 | "Artificial Intelligence Platform Architecture for Hospital Systems" Maimaitiaili | Named architecture |
| Q10 | "DeepXplainer" "Wani" "Bedi" | Metadata refinement |
| Q11 | "XAI-DR-GAN" authors | Metadata refinement |
| Q12 | "Explainable AI-driven IoMT fusion" 102472 volume | Metadata refinement |
| Q13 | site.frontiersin.org guidelines author guidelines Word template Hypothesis and Theory | Article format / template |
| Q14 | "XAI-DR-GAN" "Dwivedi" "102438" | Metadata refinement |
| Q15 | "DEPLOYR" "Corbin" "Chen" ocad114 | Metadata refinement |
| Q16 | site.nature.com "Do as AI say" | Primary human-factors evidence |
| Q17 | site.hhs.gov HIPAA treatment payment operations consent authorization research waiver | Official legal distinctions |
| Q18 | site.frontiersin.org "Frontiers_Word_Template" | Template discovery |
| Q19 | "XAI-DR-GAN" "Kashish" "sciencedirect" | Metadata refinement |
| Q20 | "Trust in automation: designing for appropriate reliance" 2004 DOI | Human-factors theory |
| Q21 | "Assurance of Machine Learning for use in Autonomous Systems" AMLAS | Assurance comparator |
| Q22 | site.sciencedirect.com/science/article "XAI-DR-GAN" "Pulkit" | Publisher metadata |
| Q23 | site.frontiersin.org "Word" "templates" "docx" | Template discovery |
| Q24 | site.hhs.gov "Research" "waiver" "Authorization" HIPAA | Official research pathway |
| Q25 | site.hhs.gov "minimum necessary" "treatment" | Legal exception verification |
| Q26 | "XAI-DR-GAN: A topology-aware explainable GAN framework for interpretable diabetic retinopathy grading" [domain filter: sciencedirect.com] | Publisher-restricted title search |
| Q27 | "Frontiers_Word_Templates.zip" | Template discovery |
| Q28 | "Frontiers" "Word templates" "zip" | Template discovery |
| Q29 | "SE-MMFusionGAN" "Dwivedi" | Metadata refinement |
| Q30 | "DEPLOYR" "Sreedevi" "Corbin" | Author-list check |
| Q31 | "10.1038/s41591-022-01772-9" | DECIDE-AI metadata |
| Q32 | "10.1136/bmj-2024-081554" | FUTURE-AI metadata |
| Q33 | "10.2196/48297" | FHIR ML review metadata |
| Q34 | "10.2196/21929" | FHIR review metadata |
| Q35 | "10.1136/qshc.2010.042085" | Sociotechnical model metadata |
| Q36 | "10.1136/amiajnl-2011-000681" | Data-quality review metadata |
| Q37 | "10.1093/jamia/ocx030" | Drift-study metadata |
| Q38 | "10.1038/s41591-019-0548-6" | Responsible-ML roadmap metadata |
| Q39 | "Model cards for model reporting" "10.1145/3287560.3287596" | Model-documentation metadata |
| Q40 | "Risk prediction models for hospital readmission" "10.1001/jama.2011.1515" | Readmission scenario source |
| Q41 | "Contemporary clinical decision support standards" "ocab070" | Clinical workflow standards |
| Q42 | site.frontiersin.org journals digital-health article-types "Hypothesis and Theory" | Final article-type verification |
| Q43 | "Safety assurance of Machine Learning for autonomous systems" "Paterson" | Assurance metadata refinement |
| Q44 | "XAI-DR-GAN" "Shariq" "Pulkit" | Author-list cross-check |
| Q45 | "Explainable AI-driven IoMT fusion" "Mamta" | Author-list cross-check |
| Q46 | "SE-MMFusionGAN" "Dwivedi" "Kajal" | Author-list cross-check |

Part B records 26 follow-up queries dated 10 September 2026, supplemented by direct URL retrievals and within-page checks. The source inventory in S7 is distinct from the number of search results or candidates considered during the original research.

## S1B: Follow-up audit queries (all dated 10 September 2026)

| **ID** | **Exact query** | **Purpose / result boundary** |
| --- | --- | --- |
| AQ01 | site.frontiersin.org journals digital health for-authors article-types Hypothesis and Theory | Journal article type |
| AQ02 | site.frontiersin.org author guidelines generative AI disclosure figures line numbers funding CRediT | Formatting/disclosure |
| AQ03 | "DeepXplainer" "107879" | Suggested paper a |
| AQ04 | "Explainable AI-driven IoMT fusion" | Suggested paper b |
| AQ05 | "XAI-DR-GAN" | Suggested paper c |
| AQ06 | "SE-MMFusionGAN" | Suggested paper d |
| AQ07 | "in-toto" "Providing farm-to-table guarantees" USENIX | Attested-step prior art |
| AQ08 | "UCONABC" usage control Park Sandhu 2004 | Continuous usage-control prior art |
| AQ09 | "clinical MLOps" architecture framework monitoring deployment healthcare | Clinical MLOps comparison |
| AQ10 | "2026" "Illinois State Academy" "Poster 27" | Prior-presentation verification |
| AQ11 | "DEPLOYR" "ocad114" | Named deployment architecture |
| AQ12 | "118th" "2026" "Illinois State Academy of Science" | Prior-presentation verification |
| AQ13 | "Operationalizing AI on Secure FHIR" "Poster" | Prior-presentation verification |
| AQ14 | "Clinical MLOps" "10.56147/aaiet.2.1.120" | Publication-version check |
| AQ15 | "Illinois State Academy" "2026" "program" | Prior-presentation verification |
| AQ16 | "Clinical MLOps" site:artificial-intelligence.engineering-technology.wren-research-journals.com | Restricted publisher retrieval |
| AQ17 | "Safety assurance of Machine Learning for autonomous systems" "111311" | Assurance-case comparison |
| AQ18 | "Do as AI say" Gaube explanations incorrect advice | Primary human-factors study |
| AQ19 | "FHIRChain" "2018" | Secure healthcare architecture |
| AQ20 | "Clinical MLOps" "Spadacini" "wren" | Publication-version refinement |
| AQ21 | "Operationalizing AI on Secure FHIR Pipelines" "2026" | Prior-presentation verification |
| AQ22 | site:myisas.org "2026" "program" | Official prior-presentation search |
| AQ23 | site:hl7.org/fhir/uv/bulkdata/STU3 "3.0.0" | Pinned Bulk Data version |
| AQ24 | site:csrc.nist.gov "800-30" "2012" | Risk method |
| AQ25 | "Lee" "See" "Trust in automation" "30392" | Appropriate-reliance source |
| AQ26 | "FUTURE-AI" "081554" | Consensus-governance source |

Direct retrieval record: official Frontiers article-type and author-guideline pages; HL7 R4/R5 Consent, Provenance, AuditEvent, RiskAssessment, R4 DocumentReference, HTTP and Attachment definitions; US Core 9; SMART 2.2; CDS Hooks 2.0.1; Bulk Data 3.0.0; NIST 800-207/207A/92 and risk-guidance record; W3C PROV-DM and Trace Context; HHS consent/authorization, research, minimum-necessary and de-identification guidance; FHIR-DHP, DEPLOYR and hospital-platform reports; named XAI publisher records; in-toto/UCON records; FHIRChain; Clinical MLOps preprint; official ISAS site. Direct DOI retrieval for Lee/See and FUTURE-AI was unsuccessful in the final pass; inherited bibliographic checks are not relabeled full-text appraisals. Some publisher openings failed or yielded abstract-only content. S7 records claim-specific limits.

## S1C: Final provenance, compliance, and bibliographic checks

Eight queries were executed on 10 September 2026 using the general web-search interface. Site restrictions below are literal query terms; no additional domain or recency filter was supplied. These checks supplement, not replace or retrospectively reclassify, S1A-B.

FQ01: site.frontiersin.org journals digital-health health-informatics article types Hypothesis and Theory

FQ02: site.frontiersin.org guidelines author-guidelines generative AI name version model source

FQ03: site.frontiersin.org policies publication ethics code availability conference abstracts CRediT

FQ04: site.myisas.org 2026 "Operationalizing" "FHIR"

FQ05: "DeepXplainer" "107879"

FQ06: "Explainable AI-driven IoMT fusion" "102472"

FQ07: "XAI-DR-GAN" "102438"

FQ08: "SE-MMFusionGAN" "01428"

Direct retrievals included the official Frontiers Health Informatics article-type page, author guidelines, and publication-ethics policy; the official ISAS 2026 annual-meeting PDF (program p. 10 and abstract pp. 28-29); GitHub v1.0.0 release metadata and tagged README, ACADEMIC_ORIGIN.md, and SECURITY.md; and the four cited publisher/PubMed records. The official program's text was accessible; its web screenshot endpoint failed. This does not establish independent observation of conference attendance. Local pre-submission implementation and absence of funding are author-confirmed facts, not facts inferred from public release timestamps. GitHub metadata identifies the public release as 9 August 2026, the evidence-bearing commit as c5c3f663cafa4d26da779f14d261e9f37d444fa4, and the tested-source commit as 71f2d9afc083ea5c0d9dad98179ed4cf8e06369f; no code was executed in this final manuscript revision.

Final bibliographic dispositions were unchanged: the healthcare/IoMT XAI review remains relevant contextual literature; the lung-cancer, retinal-grading, and MRI-CT-fusion papers remain outside the main argument on documented task relevance. PubMed/publisher abstract and metadata access, rather than complete full-text methods appraisal of all four, supports this recheck. The Springer record still identified SE-MMFusionGAN as an early accepted version. No new empirical comparison or negative feature-absence claim was introduced.

## S1D: Final editorial and source checks, 11 September 2026

Six queries were executed through the general web-search interface during the final editorial check. No domain or recency filter was passed; site terms below were literal query text. The queries did not add scientific records to the inventory.

EQ01: site.frontiersin.org guidelines author guidelines artificial intelligence name version model source acknowledgments

EQ02: site.frontiersin.org journals digital-health health-informatics for-authors article-types hypothesis theory 12000

EQ03: "DeepXplainer" "107879"

EQ04: "Explainable AI-driven IoMT fusion" "102472"

EQ05: "XAI-DR-GAN" "102438"

EQ06: "SE-MMFusionGAN" "01428"

Direct checks included the Frontiers author guidelines and Digital Health article-type guidance, HL7 R5 Provenance and R4 AuditEvent, HHS consent/authorization and minimum-necessary guidance, the publisher/PubMed records identified in S6, and the tagged v1.0.0 repository README. The XAI-DR-GAN and IoMT publisher pages could not be opened directly in this final pass, although their indexed publisher records were returned.

# Supplementary Table S2. Source-to-design traceability matrix

The 34 rows below map sources to author-paraphrased objectives, design inferences, requirements, layers and falsifiable propositions. CO1 semantic integrity; CO2 attributable identity; CO3 lawful/purpose-bounded use; CO4 isolation/containment; CO5 artifact/evidence integrity; CO6 accountable model change; CO7 detection/appropriate human response; CO8 bounded deployment. Objective assignment is retrospective interpretation, not a claim that sources use these codes. S7 provides an individual anchor and access boundary for the preceding 50 scientific references and three non-retained suggestions; three additional provenance records are separately identified there. This is conceptual traceability, not a result of a deployed assurance system.

| **Row** | **Source / evidence class** | **Extracted control objective** | **Author-derived design inference** | **R IDs** | **Layer / control point** | **Proposition / criterion and residual limit** |
| --- | --- | --- | --- | --- | --- | --- |
| S2.01 | FHIR R4; US Core; HTTP (1, 43, 45) \| S | CO1/CO5: Representation and version-addressed exchange are specified; server history retention is not guaranteed. | Require a bounded acquisition manifest and retain exact snapshots when history is insufficient. | R1, R5, R8 | L1/L2 retrieval; L4 retention | P4: reconstruction against independent snapshots; residual source truth/retention limits. |
| S2.02 | SMART; Bulk Data (2, 3) \| S | CO1/CO2/CO3: Application/backend authorization and bounded population export are interface mechanisms. | Bind delivered scope and successor use to event-specific decisions rather than treating an access token as a full use authorization. | R1-R3 | L2 request/export; L3 distributed enforcement | P1/P2: reference-denied effects and false-denial denominators; token validity does not imply lawful purpose. |
| S2.03 | CDS Hooks; standards tutorial (4, 22) \| S; T/R | CO1/CO7: Workflow interfaces and clinical reasoning standards connect information to applications. | Require patient/encounter and presentation linkage around the selected local workflow; do not presume a universal discharge hook. | R1, R6, R7 | L2 return interface; L5; Applications | P6: observed actions versus independent clinical references; vendor capability remains local. |
| S2.04 | NIST SP 800-207 (5) \| G | CO2/CO3: Access is resource/identity oriented, not inherited from network location. | Allocate a policy decision function and distributed enforcement; bind principal/delegation to material successor requests. | R2, R3 | L3 and every protected boundary | P1: independently defined over-breadth and attributable events; bad policy remains possible. |
| S2.05 | NIST SP 800-207A (6) \| G | CO2/CO4/CO8: Application and service identity support cloud-native and hybrid access control. | Treat pipelines, feature jobs and model servers as distinct actors; separate operational/analytic access paths. | R2, R4, R8 | L3 workload identity; L4 transfers | P1/P3: prohibited paths under matched local controls; shared administrators remain a bypass. |
| S2.06 | HIPAA Security Rule summary (35) \| G/L | CO4/CO5: Organizational safeguards and risk analysis apply to protected health information. | Make protection/availability responsibilities and evidence visible without claiming architecture confers legal compliance. | R4, R5, R7 | All layers; L5 risk ownership | P3/P5: recovery and incident observations; administrative/physical safeguards not inferred. |
| S2.07 | R4 Consent; HHS consent/authorization (11, 39) \| S; G/L | CO3: A consent representation is not its enforcement; consent and legal authorization have distinct roles. | Use purpose/policy authorization with consent only when applicable; record the actual operative basis. | R3 | L3 policy input; downstream gates | P2: independently adjudicated allowed/prohibited uses; local legal interpretation required. |
| S2.08 | HHS research guidance (40) \| G/L | CO3: Research authorization/waiver and other permitted pathways differ from participation consent. | Do not label all development as treatment/operations; require an approved classification and reference in the decision. | R3, R6 | L3 basis; L4 research reuse; L5 approval | P2: research/operations/waiver transition fixtures; no inferred lawful basis. |
| S2.09 | HHS minimum necessary; de-identification (41, 42) \| G/L | CO3/CO5: Minimum-necessary has exceptions; de-identification has specific requirements. | Separate architectural minimization from legal mandates and pseudonymization from established de-identification. | R3-R5 | L2 scope; L4 transformation; L5 governance | P2/P3: permitted/prohibited effects, privacy burden; no unrestricted reuse from tokenization alone. |
| S2.10 | R4 Provenance; W3C PROV-DM (10, 12) \| S | CO2/CO5: Artifacts can be related to agents, activities, sources and versions. | Construct a typed episode graph; treat a required predecessor link as a gate input where material. | R1, R5-R7 | L2/L4 producers; L5 graph | P4: independent snapshot/release reconstruction; signed false statements remain possible. |
| S2.11 | R4 AuditEvent; NIST log management (13, 23) \| S; G | CO5/CO7: Security events have identifiable actors/entities; FHIR also anticipates missing/conflicting multi-actor records. | Correlate policy and execution while keeping allow/deny distinct from technical outcome; reconcile expected events. | R2, R3, R7 | L3 event production; L5 collector | P1/P5: observer-based attribution and incident facts; logs are not their own oracle. |
| S2.12 | W3C Trace Context (27) \| S | CO5/CO7: Trace identifiers support distributed correlation. | Use case-level correlation plus unique issuer/event and typed causal links; do not treat an ID as authentication. | R5, R7 | All transition producers; L5 | P5: misleading-join and missing-link injections; privacy and collision/namespace risks. |
| S2.13 | RiskAssessment; DocumentReference (14, 44) \| S | CO1/CO5/CO6/CO7: Assessment and external artifact references have standard representations. | Link a symbolic scoring event to model/feature manifests without inventing a standard FHIR Model resource. | R1, R5, R6 | L4 score; L2/Applications return | P4/P6: output and displayed-context consistency; representation alone is not validity. |
| S2.14 | R4 Attachment.hash (46) \| S | CO5: The field specifies SHA-1 and is not a digital-signature assurance mechanism. | Keep stronger integrity data in a specified signed external manifest; do not relabel the base field as SHA-256. | R5 | L4 manifest; L5 evidence | P4: independent digest/canonicalization checks; trusted-reference/key limitations remain. |
| S2.15 | R5 Consent/Provenance/AuditEvent/RiskAssessment (47-50) \| S | CO1/CO2/CO3/CO5/CO6/CO7: Relevant field, type, cardinality, and policy representation changes are documented. | Require semantic mapping attestations and preserved source artifacts; fail unresolved restrictive mappings. | R1, R3, R5-R7 | L2 translation; L3 policy; L5 | P2/P4: restrictive-policy and replay fixtures; schema-valid translation can still lose meaning. |
| S2.16 | FHIR-DHP (15) \| E | CO1/CO8: The reported pipeline operationalizes harmonization and AI-ready export. | Retain an explicit interoperability/transform boundary, then test added policy-to-release-to-presentation linkage. | R1, R5, R8 | L2/L4 | P4: matched harmonization with/without episode links; study results do not validate this extension. |
| S2.17 | DEPLOYR (16) \| E | CO6/CO7/CO8: Reported development, live serving and monitoring connect retrospective and prospective workflows. | Use a modern control-rich comparator, not ad hoc model deployment; isolate the linkage contract. | R1, R6-R8 | L4 serving; L5 monitoring; Applications | P4-P6: matched local controls, staffing and model; unreported linkage is not assumed absent. |
| S2.18 | Hospital-platform review (19) \| R | CO1/CO6/CO7/CO8: Layered platform responsibilities overlap with integration, security and application functions. | Justify layer responsibility rather than claim a unique five-layer stack; keep consumers explicitly outside this decomposition. | R1-R8 | L1-L5; Applications outside | P1-P6: contract behavior, not layer count; no universal sufficiency inference. |
| S2.19 | FHIR implementation/ML reviews (20, 21) \| R | CO1/CO8: Published implementation work describes heterogeneous integration and reuse challenges. | Pin resource/profile/terminology context and preserve transformation evidence; avoid current adoption-rate claims. | R1, R5, R8 | L1/L2/L4 | P4: semantic and version mismatch tests; review coverage does not establish local feasibility. |
| S2.20 | Data-quality review (34) \| R | CO1: Data quality has multiple task-dependent dimensions. | Separate technical conformance from clinical fitness, retain rejects, and pin the scoring cutoff. | R1, R5, R6 | L1/L2 checks; L4 features | P4/P6: known missingness and temporal-leakage fixtures; plausibility does not prove correctness. |
| S2.21 | Model cards; responsible ML roadmap (28, 29) \| T | CO6/CO7: Documentation and lifecycle planning identify intended use, evaluation context and stakeholders. | Bind release approval and intended-use configuration to the scoring episode rather than merely storing a document. | R6, R7 | L4 registry; L5 approval | P4: prohibited-release attempts and reproducible configuration; approved models may still be unsafe. |
| S2.22 | NIST AI RMF; FUTURE-AI (7, 30) \| G | CO6/CO7/CO8: Risk governance and deployability extend beyond access control and model accuracy. | Assign monitoring interpretation, ownership, escalation and retirement to L5; keep clinical governance distinct from L3. | R3, R6, R7 | L5 with L3/L4 interfaces | P5/P6: action delay and reliance outcomes; guidance is not certification. |
| S2.23 | AMLAS (26) \| T | CO6/CO7: Assurance relates safety claims and arguments to lifecycle evidence. | Treat episode evidence as possible assurance inputs; require separate argument and independent challenge. | R5-R7 | L5 assurance ownership | P4/P5: reconstructable facts, not a claim of a completed safety case. |
| S2.24 | Sociotechnical model (37) \| T | CO7: People, workflow, technology and organizational context interact. | Include consuming workflow and human disposition without making clinical agreement a software requirement. | R6-R8 | L5 and Applications | P6: independently adjudicated action sets; interface/organization effects may dominate. |
| S2.25 | Appropriate-reliance theory; clinical advice experiment (31, 32) \| R/T; E | CO7: Reliance should fit system capability; misleading advice can influence decisions. | Test incorrect and out-of-scope advice and both over- and under-reliance; do not optimize self-reported trust. | R6, R7 | Applications; L5 human-factors evaluation | P6: unsafe following/appropriate-action denominators; polished-provenance harm remains a hypothesis. |
| S2.26 | XAI/IoMT review (33) \| R | CO7: Explanation methods concern model interpretation and deployment challenges. | Separate explanation artifacts from authorization/provenance evidence; no inferred clinician-reliance benefit. | R6, R7 | L4 explanation context; Applications | P6: evaluate behavior rather than assume explanatory detail improves decisions. |
| S2.27 | Calibration drift study (36) \| E | CO6/CO7: Calibration can change over time in its reported AKI setting. | Monitor calibration when labels exist, but do not equate drift alarms with harm or transfer effect sizes to readmission. | R6, R7 | L5 model monitoring | P5: known injected change; future pilot calibration with label-delay reporting. |
| S2.28 | Readmission review (51) \| R | CO7/CO8: Historical models differed in purpose, timing and performance. | Use a bounded discharge-planning example, explicitly distinguish prediction from intervention benefit and avoid future-feature leakage. | R1, R6-R8 | L4 cutoff; Applications; L5 | P6 and later clinical evaluation; no model choice/effectiveness claim from this illustration. |
| S2.29 | NIST risk-assessment guidance (38) \| G | CO4/CO7: Risk reasoning should make threat conditions and consequence assumptions explicit. | State conditional qualitative priorities with separate uncertainty and residual mechanisms, not fabricated probabilities. | R2-R7 | All layers; L5 risk owners | P1-P5: scenario selection and independent incident truth; priorities require local reassessment. |
| S2.30 | DECIDE-AI (52) \| G | CO7/CO8: Early live clinical AI evaluation has reporting requirements including context and human factors. | Reserve live-pilot reporting for future work after lower-risk gates; expert review and synthetic testing are not clinical validation. | R7, R8 | L5 evaluation and expansion gates | P6/Stage 4: workflow evidence; Stage 5 separately tests patient outcomes. |
| S2.31 | UCONABC (24) \| T | CO2/CO3: Authorizations, obligations, conditions and mutable state support ongoing usage control. | Do not claim ongoing authorization as novel; specialize its policy dependency to the clinical episode. | R2/R3 | L3 and successor gates | P1/P2: independent policy fixtures; equivalent usage-control implementation limits novelty. |
| S2.32 | in-toto (25) \| T/E | CO5/CO6: Signed step and artifact relationships enable integrity verification. | Credit cross-stage attestation; bind clinical purpose and presentation/disposition as additional typed context. | R5/R6/R7 | L4/L5 evidence and release gates | P4/P5: correct independent reconstruction; verified bytes do not establish truthful input or clinical safety. |
| S2.33 | FHIRChain (17) \| T/E | CO1/CO2/CO3: Secure FHIR-oriented clinical sharing uses identity and access mechanisms. | Treat secure sharing as a capable predecessor, not missing security; compare actual downstream episode linkage. | R1/R2/R3/R5 | L2/L3/L4 data-sharing boundary | P1/P2/P4: no unsupported absence claims or empirical superiority. |
| S2.34 | Clinical MLOps (18) \| T; accessible preprint | CO3/CO5/CO6/CO7: Runtime purpose checks, versioned inference lineage, clinical observability and human oversight. | Narrow novelty to the explicit joint prerequisite/expectation contract; substantial overlap acknowledged. | R3/R5/R6/R7 | L3/L4/L5 and Applications | P2/P4/P5/P6: retain native safeguards; no borrowing of reported outcomes or legal generalizations. |

# Supplementary Table S3. Detailed asset-centered threat mapping

The T identifiers refer to the conditional priorities in main Table 3. No local hospital asset inventory, incident frequency or measured control effectiveness was used. Table 3's priority rules and uncertainty definitions control interpretation. These controls and evidence are proposed for implementation and testing, not asserted to exist.

| **ID** | **Assets** | **Actors / failure sources** | **Surface / misuse path** | **Primary controls** | **Independent reference / evidence** | **Residual mechanism** |
| --- | --- | --- | --- | --- | --- | --- |
| T1 | Patient data; identity/delegation; policy attributes | Stolen valid credentials; insider; compromised application; stale context | FHIR/SMART/service-to-service requests; broader successor purpose or scope | Unique identities and scoped tokens in both conditions; M1 checks predecessor identity/purpose binding. | Independent policy fixtures and effect observations; decision and execution records. | Valid-session abuse and a wrong approved policy remain. A denied lawful request is a measured harm. |
| T2 | Operational FHIR service; analytic copies; backups; identity/evidence dependencies | Ransomware; cloud/configuration failure; shared administrator | Permitted transfer abused; recovery path bypass; unavailable common services | Separate administration/transfer paths, bounded egress, tested recovery and ordinary-care fallback; M1 binds manifests. | Independent asset inventory/probes; transfer acknowledgments; recovery/containment timelines. | Authorized exfiltration, shadow copies or common-admin compromise may bypass the design. Dependency failures can delay care. |
| T3 | Source resources, profiles, terminology, feature definitions/values | Local source change; transformation error; delayed or implausible input | FHIR-to-analytic mapping; feature windows; training/serving mismatch | Version/cutoff manifest, reject handling and reference compatibility; independently curated test fixtures. | Preserved source/feature snapshots; known mismatch/temporal-leakage injections. | Conformance cannot establish source truth. Incorrect but consistently versioned data can pass all checks. |
| T4 | Model binaries, container/dependencies, registry, approval and configuration | Build/registry compromise; supply chain; unreviewed release | CI/CD and model serving; stale approval reused on different target | Digest and approval checks, separation of duties and rollback; M1 binds score to compatible release evidence. | Controller-held approval/artifact fixtures; deployment logs; score/release links. | Compromised signing keys and unsafe approved models remain possible. Integrity and reproducibility do not prove clinical quality. |
| T5 | Scoring output, patient/encounter context, clinical presentation/action | Output tampering/replay; context error; automation bias | Return interface; SMART/CDS integration; Task creation; clinical interpretation | Destination/context gate, recency and presentation linkage; meaningful human choice with bounded return. | Independent observer and clinical reference; display/action records. | A polished but misleading result may increase unsafe following. Disagreement is not automatically an incident. |
| T6 | Evidence collector, graph, incident console and retained manifests | Producer/collector compromise; dropped records; clock error; alert overload | Outbox/forwarder, evidence store, cross-domain joins, analyst workflow | Independent expected-event state model, integrity-protected retention, acknowledgment reconciliation and alert ownership. | External event schedule; expected/observed reconciliation; protected incident facts and action times. | Pre-capture loss and forged producer evidence may persist. Evidence concentration increases privacy and breach consequences. |
| T7 | Optional external device/IoMT observations | Untrusted device, quality drift or unsupported interface | A newly introduced device-data source, outside the baseline scoring assumption | New interface-specific risk and quality assessment; identity, source representation and transition controls before extension. | Future device provenance/quality fixtures, not data asserted here. | The base architecture does not demonstrate sufficiency for an unassessed new device/source. |

Residual-risk responsibilities and review triggers are proposed as follows. T1: identity and privacy owners, following changes in roles, purposes, or exceptions. T2: operations and security owners, following topology, dependency, or recovery changes. T3: data and clinical owners, following profile, terminology, feature, or intended-use changes. T4: model-release and security owners, following artifact, dependency, or approval changes. T5: clinical-informatics owners, following destination, interface, or encounter mismatches. T6: security and records/privacy owners, following capture loss, collector or key changes, or alert overload. T7: device, data, and clinical owners before an external interface is enabled. These are responsibilities to assign, not appointed individuals. Local evidence is required to reassess residual priority.

# Supplementary Table S4. FHIR evidence contract and cross-version preservation

The context is FHIR R4 4.0.1, with US Core 9.0.0 used only for applicable clinical resource profiles. The evidence resources use base definitions plus the described local conceptual conventions. The identifiers below are symbols, not a JSON Bundle, validated StructureDefinition, working implementation guide, patient dataset, model result or actual approval. R5 is discussed as a translation target; compatibility is conditional.

| **Contract item** | **Representation / linkage** | **Preservation condition and limitation** |
| --- | --- | --- |
| S4.1 Episode identity | S is an issuer-namespaced episode identifier; each PD, acquisition, transform, score, U and H also has its own event ID. | Local contract, not a claim that every FHIR resource has an identifier field. A shared trace ID is useful but is not an authenticator or a substitute for typed causal references. (27) |
| S4.2 Policy-to-source binding | PD-read binds I, purpose, patient/encounter scope, action, destination, policy/basis version, applicable C and obligations before read/prefetch. D records actual delivered scope and versions. | Gate separately records policy decision and execution disposition. C is omitted with an explicit basis explanation when consent is not operative. Unknown is not permit. (11, 39, 40) |
| S4.3 Source-to-feature binding | D contains namespaced historical resource references or an acquisition snapshot; F identifies definition/code/terminology/input cutoff and values used. | Feature definition and feature values are different artifacts. No actual data values or patient resource instances are supplied. Expected inputs/rejects must be retained; unavailable inputs are not silently complete. (34, 45) |
| S4.4 Feature/model/release-to-score | PD-score checks F compatibility with M and A. S records model digest, runtime/configuration, threshold version, input cutoff and output reference. | Registry presence does not imply approval. A names the approved target/use and current state. P4 tests replay against controller-held artifacts, not the system's own history alone. (14, 28) |
| S4.5 Provenance and audit | Provenance targets RiskAssessment/score-S/_history/1 (symbolic). Its source entity references point to D/F/M/A manifests as appropriate. AuditEvent.entity.what links the score or manifest; detail stores local episode/decision keys. | R4 Provenance.recorded is required. AuditEvent.id is a resource ID, not a business identifier. R4 entity.detail.type/valueString conventions need local documentation. Do not overwrite the event's technical outcome with a policy verdict. (12, 13) |
| S4.6 External integrity manifest | DocumentReference.content.attachment.url points to a controlled manifest containing artifact identity, digest algorithm, canonicalization, signature/key identity and predecessor links. | R4 Attachment.hash is SHA-1; no SHA-256 substitution is made. Retain signing/verification context and access-controlled source bytes. Hashes and signatures do not prove the producer's clinical assertions. (44, 46) |
| S4.7 Presentation/action binding | PD-return checks patient/encounter/destination/freshness. U binds displayed content/version to S and an authorized session. H identifies its U and documented human disposition. | Record pending, not captured and not applicable separately. Viewing does not establish comprehension; no action does not establish rejection; disagreement does not establish a technical failure. (4, 37) |
| S4.8 R4/R5 Consent | Map R4 patient to R5 subject only when the patient meaning is preserved. Preserve scope, policy, provision restrictions, and statuses rather than renaming fields mechanically. Distinguish original creation time from the R5 execution date. | Use fixtures for permit/deny effect, nested restrictions, purpose, recipient, period, data scope and basis. Record unresolved mappings. R5 regulatoryBasis/policyBasis/decision cannot be populated by guessing from a text label. (11, 47) |
| S4.9 R4/R5 Provenance | Preserve the required local recording time even though R5 allows omission. Map R4 reason to R5 authorization according to CodeableReference semantics, retaining versioned targets, entities, and appropriate agent roles. | R5 entity roles remove derivation and add instantiates; do not treat those as interchangeable. The illustrative source role is common to both releases. Additional R5 patient/encounter/basedOn does not replace original entity links. (12, 48) |
| S4.10 R4/R5 AuditEvent | Map event classification and occurrence deliberately: type/subtype vs category/code, period vs occurred[x], simple vs structured outcome, purposeOfEvent vs authorization. | Retain original code systems, actor/source attribution, recording time, and local episode/decision key meanings. A structurally valid transformed event may still describe a different outcome. (13, 49) |
| S4.11 R4/R5 RiskAssessment | Preserve identifier, patient/encounter, horizon, occurrence, basis and scorer/release links; map reasonCode/reasonReference into reason CodeableReference without losing the original meaning. | Broader performer targets in R5 do not justify silently replacing a Device scorer. A new resource version or translated representation requires a recorded transformation edge. (14, 50) |
| S4.12 Translation loss and oracle | Keep both source and translated objects, mapping/profile/terminology versions, source/target identifiers and an integrity attestation. Test with independent policy and semantic fixtures. | A lost restriction, missing required timestamp, unresolved namespace, discarded source bytes, or invalid inherited signature leaves continuity unresolved or broken. No generic bidirectional R4/R5 compatibility claim is made. (47-50) |

## Illustrative non-FHIR event envelope (notation, not executed software)

E-score = {episode: S; eventId: issuer-scoped scoring-event; predecessors: [PD-score, D, F, M, A]; resultRef: RiskAssessment/score-S/_history/1; eventTime: t-score; recordedAt: t-capture; producerRef: I; integrityRef: signed-manifest; disposition: explicit execution state}. The integrity manifest specifies algorithm, canonicalization, expected digest, producer key and verification context. PD-score separately records the policy verdict and its reasons. U refers to S; H refers to U. An independent expected-event/state model, not this record itself, establishes which links are required for that path.

Delegation translation rule: Provenance.agent.onBehalfOf permits Device/RelatedPerson in R4 but not R5. Preserve the source reference, role, issuer and versioned delegation chain through a versioned external delegation attestation while keeping the R5 element conformant. A permitted target type is not a license to invent an organizational/practitioner identity. Unresolved loss blocks a dependent protected transition. Source recording time, source bytes and the mapping attestation remain separately identifiable.

# Supplementary Table S5. Future proposition test cards

## Common decision rules

All cards are future study designs, not studies performed. Exactly one primary endpoint is specified per proposition. Support requires benefit beyond a prospectively set meaningful margin and all harm bounds met; secondaries cannot rescue a failed primary. Adequately precise equivalence/reversal or breached harm bounds are counterevidence. Low precision, disputed references, absent opportunities or unmatched conditions are inconclusive. Native safeguards in a modern comparator remain intact. Link-removal ablations are confined to synthetic/simulated designs and labeled degraded conditions. No effect margin, sample size or result is fabricated. Missing, failed, withheld and undetected cases are retained as specified below.

## P1: Cross-boundary unauthorized effects

**Primary prohibited-effect rate:** test requests producing at least one fixture-prohibited effect divided by all test requests attempting at least one prohibited effect. This includes over-broad requests that return forbidden fields alongside permitted content. Over-breadth means any resource, field, or action outside the approved fixture scope. False-denial rate: reference-permitted requests incorrectly denied divided by all reference-permitted requests. Correct attribution is assessed against all independently observed events, including denials.

**Independent reference:** Independent actor/scope policy fixtures; harness observes actual effects including denials/failures.

**Matched contrast / ablation:** Matched unique IDs, token duration, policies, workloads, latency budget; ablate predecessor identity/delegation link only.

**Adverse outcomes:** False denials / reference-permitted attempts; request latency distribution; failed workflow count; authentication and evidence-privacy burden.

## P2: Policy-discordant downstream reuse

**Primary:** completed reference-prohibited uses/all prohibited-use attempts. Secondary: policy-change-to-sustained-concordance delay; never-enforced changes separately disclosed. False denial and care delay are harm gates.

**Independent reference:** Independent state machine for TPO, approved research, consent where applicable, withdrawal, expiry, lawful retention and authorized exceptions.

**Matched contrast / ablation:** Identical current local policy engines and fixtures; ablate purpose/basis/version link, not the engine.

**Adverse outcomes:** False denials / permitted attempts; care/processing delay; exception misuse; retained consent/purpose metadata burden.

## P3: Prohibited operational/analytic transfers

**Outcome and denominator:** Successful prohibited transfers / all prohibited-transfer attempts. Secondary: unauthorized reach / enumerated assets probed; containment time with uncontained trials retained.

**Independent reference:** Controller-held inventory, permissions, attack routes/credentials and external effect/recovery observations.

**Matched contrast / ablation:** Equivalent segmentation and administrators; ablate transfer-manifest dependency, holding probes/privileges and recovery staff constant.

**Adverse outcomes:** Extra connector outages; recovery delay; operational latency; staff cost; common policy/evidence service dependence.

## P4: Correctly reconstructable episodes

**Primary correct-reconstruction rate:** episodes matching every prespecified required controller-held reference within a fixed investigation budget divided by all sampled episodes. The required facts cover identity, policy, source, feature, model/release, score, presentation, and disposition. Branch-specific pending or not-applicable states must match the independent expected record. Any missing critical reference or incorrect join fails the episode. Failed, missing, and lawfully inaccessible reconstructions remain in the feasibility denominator. Replay requires exact equality in a specified deterministic environment or a justified preregistered tolerance and decision category for nondeterministic execution. Replay is secondary, not a substitute for correct reconstruction.

**Independent reference:** the controller independently records identity and policy references, source and feature snapshots, digests, release fixtures, runtime configuration, original outputs, presentation, and disposition before mismatch injection or reconstruction. The complete reference episode is withheld from reconstruction operators.

**Matched contrast and ablation:** preserve equivalent feature and model versioning and release approvals. Separately remove the source-to-feature link or release-to-score link only in a labeled simulated ablation.

**Adverse outcomes:** Serving delay, reconstruction time, retained sensitive data/storage cost, unavailable lawful historical access, prohibited-release effects.

## P5: Detection delay with incident coverage

**Primary detection delay** is the mean time to independently adjudicated detection, capped at a common prespecified observation horizon, tau, across all seeded incidents. Undetected incidents receive tau; incidents that end earlier are retained. Detection coverage is the number detected divided by all seeded incidents. Unexpected observation loss requires a disclosed sensitivity analysis or an inconclusive comparison. Fact reconstruction and action latency are secondary.

**Independent reference:** Concealed injection schedule and fixed required incident-fact set; independent adjudication of alerts and response.

**Matched contrast / ablation:** Equivalent multichannel monitors, thresholds, training, incident severity and analysts; ablate cross-domain link or expectation reconciliation.

**Adverse outcomes:** False alerts per monitored hour and episode; analyst time; delayed response to other events; wrong joins; evidence-store privacy concentration.

## P6: Unsafe following and appropriate clinical action

**Primary:** independently observed decisions in the blinded panel's acceptable action set/all adjudicated assigned cases, including withheld/unavailable advice; stratify by advice condition. Unsafe following = inappropriate model-consistent actions/all cases with adjudicated inappropriate advice. Under-reliance = no acceptable indicated action/all cases needing such action. Legitimate clinical disagreement is not an error.

**Independent reference:** Blinded independent clinical-panel acceptable action sets; independent session observation; model output is not the oracle.

**Matched contrast / ablation:** same underlying clinical advice, comparable users/training, equally usable override interfaces and matched polish. Use closed vignette simulation for knowingly incorrect/incompatible advice, not patient care. Remove warnings only in that protected simulation; include withheld cases in the primary denominator.

**Adverse outcomes:** Unsafe following, inappropriate rejection, interpretation time, alert/documentation burden, accessibility, self-confidence without behavioral improvement.

# Supplementary Table S6. Verification and disposition of reviewer-suggested XAI papers

Each suggested publication was assessed using the accessible material identified below. The healthcare/IoMT review is retained for its explanation-method context. Three task-specific papers are not included in the main bibliography because their reported questions do not directly support the proposed authorization and evidence contract. These decisions concern relevance, not study quality or the absence of unreported controls. The final checks on 11 September 2026 reconfirmed the available bibliographic records; restricted access remains explicit.

## Item 1 (R2.2a): DeepXplainer: An interpretable deep learning based approach for lung cancer detection using explainable artificial intelligence

Wani NA, Kumar R, Bedi J. DeepXplainer: An interpretable deep learning based approach for lung cancer detection using explainable artificial intelligence. Comput Methods Programs Biomed. (2024) 243:107879. doi: 10.1016/j.cmpb.2023.107879.

**Access and verification:** the recorded PubMed and publisher abstracts identify the title, authors, January 2024 issue, article number, and DOI. These metadata were reconfirmed through indexed primary-source records on 11 September 2026. The 2023 online publication history is distinguished from the 2024 issue year. The full article was not appraised.

**Disposition:** Not added to the manuscript reference list. The indexed abstract describes a task-specific predictive/explanation approach using the Survey Lung Cancer dataset. It is not an evaluation of FHIR policy/evidence linkage or clinician reliance. Adding it would not substantiate the architectural claim. This is a relevance decision, not a criticism of study quality.

Record: https://pubmed.ncbi.nlm.nih.gov/37897989/

## Item 2 (R2.2b): Explainable AI-driven IoMT fusion: Unravelling techniques, opportunities, and challenges with Explainable AI in healthcare

Wani NA, Kumar R, Mamta, Bedi J, Rida I. Explainable AI-driven IoMT fusion: Unravelling techniques, opportunities, and challenges with Explainable AI in healthcare. Inf Fusion. (2024) 110:102472. doi: 10.1016/j.inffus.2024.102472.

**Access and verification:** the recorded publisher abstract, introduction, and section previews support the scope assessment. Indexed publisher metadata reconfirmed the author order, including Mamta, the year, volume, article number, and DOI on 11 September 2026. Direct full-page access failed during that final check. No complete full-text appraisal is claimed.

**Disposition:** retained for healthcare/IoMT explanation-method context. The distinction between explanatory material and operational authorization/provenance is an architectural interpretation in Section 3.3, not a claim that this review tests the proposed contract. It is not evidence that polished explanations or provenance displays improve clinical reliance.

Record: https://www.sciencedirect.com/science/article/abs/pii/S1566253524002501

## Item 3 (R2.2c): XAI-DR-GAN: A topology-aware explainable GAN framework for interpretable diabetic retinopathy grading

Dwivedi P, Chakraborty S, Wani NA, Wani MA, Bashir S, Shakil KA. XAI-DR-GAN: A topology-aware explainable GAN framework for interpretable diabetic retinopathy grading. Eng Sci Technol Int J. (2026) 80:102438. doi: 10.1016/j.jestch.2026.102438.

**Access and verification:** the recorded author-list checks and indexed publisher abstract identify the title, DOI, volume, and task. The publisher search record was reconfirmed on 11 September 2026; direct full-page access failed. The earlier author-list check is retained as a separate verification step, not represented as a full-text appraisal.

**Disposition:** Not added to the manuscript reference list. The reported task is retinal grading/augmentation with topology-aware generative modeling and explanation. This does not directly test cross-boundary authorization, release evidence, or human reliance. No absence of security controls in the authors' broader system is inferred.

Record: https://www.sciencedirect.com/science/article/pii/S2215098626001643

## Item 4 (R2.2d): SE-MMFusionGAN: A Sustainable and Explainable Multi-modal Fusion GAN for MRI-CT Image Fusion and Anomaly Localization

Dwivedi P, Chakraborty S, Wani NA, Kajal M, Gaur K. SE-MMFusionGAN: A Sustainable and Explainable Multi-modal Fusion GAN for MRI-CT Image Fusion and Anomaly Localization. Int J Comput Intell Syst. (2026), accepted early-online article; no volume/page data asserted. doi: 10.1007/s44196-026-01428-3.

**Access and verification:** the publisher page retrieved on 11 September 2026 identifies an accepted early version published online on 27 June 2026, with the stated authors and DOI. The accessible abstract and appendix support the scope assessment. The complete main methods were not available in the retrieved HTML, and no unverified volume or page information is supplied.

**Disposition:** Not added to the manuscript reference list. Its MRI-CT fusion/anomaly-localization and efficiency/explanation objectives concern a model-level method, not the proposed policy-to-workflow evidence contract. It does not substantiate a claim of appropriate clinical reliance in this use case.

Record: https://link.springer.com/article/10.1007/s44196-026-01428-3

The recorded author publication pages used for cross-checking the 2026 metadata were https://sites.google.com/site/drshariqbashir/selected-publications and https://sites.google.com/view/soumenduchakraborty/publication. Author-reported metadata is not an independent appraisal of inaccessible full text. Publication status is reported as encountered in the dated checks.

# Supplementary Table S7. Bounded source inventory, access limits and individual rationale anchors

The documented inventory contains 56 distinct records: 53 main references and three reviewer-suggested task-specific papers assessed in S6 but not retained. The fourth suggested publication was retained and appears above as record 33 (IOMT); the excluded-record labels accordingly run XAI-a, XAI-c, and XAI-d. The main references comprise 50 scientific, standards, or guidance sources and three provenance records for the academic origin, versioned software, and prior conference abstract. These are inventory and disposition counts, not original search-hit or screening denominators. Author-confirmed local development is distinguished from public artifact dates. Reference numbering matches the main manuscript. All control-objective, requirement, layer, and proposition assignments are author-derived; a source may inform a boundary assumption, comparison, or provenance statement without supporting the direction of a proposition.

| Record / main reference | **Source** | **Exact anchor / access boundary** | **Retention / exclusion rationale** | **Design mapping: objective; requirement; layer; proposition** |
| --- | --- | --- | --- | --- |
| 1. FHIR4 | FHIR Release 4, version 4.0.1: overview | Overview; resources and conformance Official versioned page | FHIR representation supplies CO1, not proof of source truth. | CO1/CO5; R1/R5; L1/L2/L4; P4 |
| 2. SMART | SMART App Launch Implementation Guide, version 2.2.0 | Scopes and backend services Official versioned page | Application/workload authorization, not a complete lawful-use decision. | CO2/CO3; R1/R2/R3; L2/L3; P1/P2 |
| 3. BULK | FHIR Bulk Data Access Implementation Guide, version 3.0.0 | STU3 English Home; Conformance; pre-authorized client Official versioned page; 2025-12-11 version date | Population export makes delivered-scope boundaries relevant. | CO1/CO3; R1/R3; L2/L3; P1/P2 |
| 4. CDS | CDS Hooks specification, version 2.0.1 | Exchange; hookInstance; cards/feedback Official versioned page | Workflow mechanisms require separate encounter and presentation evidence. | CO1/CO7; R1/R7; L2/L5/Applications; P6 |
| 5. ZT | Zero trust architecture | SP 800-207, §2.1 tenets; §3 logical components Official full publication | Identity/resource policy supports bounded requests; not AI clinical governance. | CO2/CO3; R2/R3; L3; P1/P2 |
| 6. ZTA | A zero trust architecture model for access control in cloud-native applications in multi-location environments | SP 800-207A, cloud-native access-control model Official full publication | Service identities and distributed access inform hybrid separation. Title corrected to Multi-Location. | CO2/CO4/CO8; R2/R4/R8; L3/L4; P1/P3 |
| 7. AIRMF | Artificial Intelligence Risk Management Framework (AI RMF 1.0) | AI RMF: Govern, Map, Measure, Manage Supplied official-source check retained; not fresh full-text appraisal | Lifecycle risk functions motivate accountable change/monitoring. | CO6/CO7; R6/R7; L4/L5; P4/P5/P6 |
| 10. PROVDM | PROV-DM: the PROV data model | PROV-DM entity/activity/agent and relations Official full Recommendation | Typed lineage represents derivation, not authorization or clinical truth. | CO5; R5/R6; L4/L5; P4 |
| 11. CONS4 | Consent resource: FHIR Release 4, version 4.0.1 | Consent scope/use and resource structure Official R4 definition | Consent represents rules where applicable; no policy engine implied. | CO3; R3; L3; P2 |
| 12. PROV4 | Provenance resource: FHIR Release 4, version 4.0.1 | Provenance target, agent, entity, recorded Official R4 definition | Versioned artifact origin/actors support reconstructability. | CO2/CO5; R2/R5/R6; L4/L5; P4 |
| 13. AUD4 | AuditEvent resource: FHIR Release 4, version 4.0.1 | AuditEvent scope, entity, outcome Official R4 definition | Security/operational events are distinct from policy decisions. | CO5/CO7; R5/R7; L3/L5; P1/P5 |
| 14. RISK4 | RiskAssessment resource: FHIR Release 4, version 4.0.1 | RiskAssessment identifier, basis, performer, prediction Official R4 definition | Represent the assessment and clinical context, not an invented model registry. | CO1/CO6/CO7; R1/R6/R7; L4/Applications; P4/P6 |
| 15. DHP | A standardized clinical data harmonization pipeline for scalable AI application deployment (FHIR-DHP): validation and usability study | Abstract; five-step pipeline; reported evaluation Full JMIR article | Credit implemented harmonization/export; report scope is not feature absence. | CO1/CO8; R1/R8; L2/L4; P4 |
| 16. DEPLOYR | DEPLOYR: a technical framework for deploying custom real-time machine learning models into the electronic medical record | DEPLOYR development, serving and monitoring modules Full PMC article | A strong clinical deployment comparison, not an opaque-score baseline. | CO6/CO7/CO8; R6/R7/R8; L4/L5/Applications; P4/P5/P6 |
| 17. FHIRCHAIN | FHIRChain: Applying blockchain to securely and scalably share clinical data | FHIRChain design: identity, tokens and clinical sharing Full PMC primary article | Secure healthcare predecessor, with reported scope credited. | CO1/CO2/CO3; R1/R2/R3/R5; L2/L3/L4; P1/P2/P4 |
| 18. CLINICALMLOPS | Clinical MLOps: A framework for responsible deployment and observability of AI systems in cloud-native healthcare platforms | Clinical MLOps §§3.1-3.4: purpose, audit/lineage, monitoring, oversight Accessible full preprint; linked journal version not retrieved | Substantial overlap narrows novelty. Neither reported metrics nor legal claims are adopted. | CO3/CO5/CO6/CO7; R3/R5/R6/R7; L3/L4/L5/Applications; P2/P4/P5/P6 |
| 19. HOSP | Artificial intelligence platform architecture for hospital systems: systematic review | Results: infrastructure/data/algorithm/application/security-compliance layers Full JMIR article | Alternative five-layer taxonomy disproves layer count as novelty. | CO1/CO6/CO7/CO8; R1/R6/R7/R8; L1-L5; P4/P5/P6 |
| 20. AYAZ | The Fast Health Interoperability Resources (FHIR) standard: systematic literature review of implementations, applications, challenges and opportunities | Review abstract: implementation challenges Supplied review-source check retained; no fresh full-text appraisal | Interoperability context only, not observed contract benefit. | CO1; R1; L2; P4 |
| 21. BALCH | Machine learning-enabled clinical information systems using Fast Healthcare Interoperability Resources data standards: scoping review | Scoping review: ML-enabled FHIR systems Supplied review-source check retained; no fresh full-text appraisal | FHIR-enabled ML context; no blanket absence of security inferred. | CO1/CO8; R1/R8; L2/L4; P4 |
| 22. CDSREVIEW | Contemporary clinical decision support standards using Health Level Seven International Fast Healthcare Interoperability Resources | FHIR clinical decision-support standards and interfaces Supplied source check retained; no fresh full-text appraisal | Standards define integration options, not appropriate human reliance. | CO1/CO7; R1/R7; L2/Applications; P6 |
| 23. LOG | Guide to computer security log management | SP 800-92: log management and operational process Official full publication | Logging can be sophisticated; additional contract must be explicit. | CO5/CO7; R5/R7; L5; P4/P5 |
| 24. UCON | The UCONABC usage control model | Abstract: ABC, obligations/conditions, continuity and mutability ACM primary abstract/record | Continuous usage control is prior art; retained as a novelty challenge. | CO2/CO3; R2/R3; L3; P1/P2 |
| 25. INTOTO | in-toto: Providing farm-to-table guarantees for bits and bytes | USENIX abstract: signed step/artifact guarantees Primary conference paper record | Attested workflow linkage is prior art; no clinical safety extrapolation. | CO5/CO6; R5/R6/R7; L4/L5; P4/P5 |
| 26. AMLAS | Safety assurance of Machine Learning for autonomous systems | AMLAS abstract, lifecycle assurance method Publisher abstract/preview and institutional record | Assurance argument differs from retained runtime evidence. | CO6/CO7; R6/R7; L5; P4/P6 |
| 27. TRACE | Trace Context, version 1 | Trace Context: traceparent/tracestate and security considerations Official full Recommendation | Correlation propagates context but does not establish permission. | CO5/CO7; R5/R7; L3-L5; P4/P5 |
| 28. CARDS | Model cards for model reporting | Model cards: intended use, evaluation, limitations Supplied source check retained; no fresh full-text appraisal | Documentation motivates release context, not clinical validity. | CO6; R6; L4/L5; P4/P6 |
| 29. WIENS | Do no harm: a roadmap for responsible machine learning for health care | Roadmap for responsible ML: deployment context and monitoring Supplied source check retained; no fresh full-text appraisal | Clinical lifecycle concerns bound a secure model's acceptable use. | CO6/CO7; R6/R7; L4/L5; P4/P5/P6 |
| 30. FUTURE | FUTURE-AI: international consensus guideline for trustworthy and deployable artificial intelligence in healthcare | FUTURE-AI consensus guidance Inherited record; current DOI fetch failed; correction record checked | Consensus context only. No new full-text verification claim. | CO6/CO7/CO8; R6/R7/R8; L5; P4/P5/P6 |
| 31. RELIANCE | Trust in automation: designing for appropriate reliance | Trust in automation: appropriate reliance Inherited primary citation; current DOI fetch failed | Conceptual reliance framing; no framework-specific effect claimed. | CO7; R7; L5/Applications; P6 |
| 32. GAUBE | Do as AI say: susceptibility in deployment of clinical decision-aids | Experimental advice study, abstract and full article Primary Nature/PubMed article | Incorrect advice can impair decisions; not a provenance-polish experiment. | CO7; R7; Applications; P6 |
| 33. IOMT | Explainable AI-driven IoMT fusion: Unravelling techniques, opportunities, and challenges with Explainable AI in healthcare | IoMT XAI review: abstract, introduction, section previews Publisher previews; not complete full-text appraisal | Retain XAI context; authorization/explanation distinction is our inference. | CO7; R7; Applications; P6 |
| 34. QUALITY | Methods and dimensions of electronic health record data quality assessment: enabling reuse for clinical research | Data quality dimensions for EHR reuse Supplied primary-source check retained; no fresh full-text appraisal | Conformance is not truth; missingness/currency remain task-dependent. | CO1; R1/R5; L1/L2/L4; P4 |
| 35. HIPAA | Summary of the HIPAA Security Rule | HHS Security Rule safeguards overview Official guidance record | Security safeguards motivate protection, not complete legal compliance. | CO4/CO5; R4/R5; L3/L4; P3/P4 |
| 36. DRIFT | Calibration drift in regression and machine learning models for acute kidney injury | Calibration drift in AKI models Supplied empirical-source check retained; no fresh full-text appraisal | Supports possibility of drift, not measured drift in this design. | CO6/CO7; R6/R7; L5; P5 |
| 37. SOCIO | A new sociotechnical model for studying health information technology in complex adaptive healthcare systems | Sociotechnical model for health information technology Supplied primary-source check retained; no fresh full-text appraisal | Workflow/people/context motivate explicit human and operational controls. | CO7; R7; L5/Applications; P6 |
| 38. RISKGUIDE | Guide for conducting risk assessments | SP 800-30r1 abstract and NIST risk-method description Official record and risk guidance; no local risk observations | Conditional prioritization includes uncertainty, not invented frequencies. | CO4/CO7; R4/R7; L3-L5; P3/P5 |
| 39. CONSENTLAW | What is the difference between consent and authorization under the HIPAA Privacy Rule? | HHS FAQ 264: consent versus authorization Official guidance full page | TPO consent can be voluntary; authorization and other permitted bases differ. | CO3; R3; L3; P2 |
| 40. RESEARCHLAW | Research | Research: authorization and waiver pathways Official guidance full page | Research pathways are distinct from treatment/participation consent. | CO3; R3; L3; P2 |
| 41. MINNEC | Minimum necessary | Minimum Necessary: exceptions and implementation Official guidance full page | Architectural minimization is not a claim that every HIPAA use shares one rule. | CO3/CO5; R3/R5; L2/L3; P2 |
| 42. DEID | Guidance regarding methods for de-identification of protected health information in accordance with the HIPAA Privacy Rule | De-identification methods and identifiers Official guidance full page | Pseudonymization/tokenization alone do not establish legal de-identification. | CO3/CO5; R3/R5; L2/L4; P2/P4 |
| 43. USCORE | US Core Implementation Guide, version 9.0.0 (STU 9) | US Core STU9 homepage and FHIR dependency Official versioned guide | R4-based clinical profiles do not profile every evidence resource. | CO1; R1; L2; P4 |
| 44. DOCREF | DocumentReference resource: FHIR Release 4, version 4.0.1 | DocumentReference content and context Official R4 definition | Pointers to external artifacts do not invent a FHIR Model registry. | CO5/CO6; R5/R6; L4/L5; P4 |
| 45. HTTP | FHIR Release 4, version 4.0.1: RESTful API | RESTful API: versions/history and references Official R4 page | Version-addressed resources need available history or retained snapshots. | CO1/CO5; R1/R5; L2/L4; P4 |
| 46. ATTACH | FHIR Release 4, version 4.0.1: Attachment detailed definitions | Attachment.hash definition Official R4 datatype definition | R4 hash is SHA-1; modern digest must not be mislabeled in that field. | CO5; R5; L4/L5; P4 |
| 47. CONS5 | Consent resource: FHIR Release 5, version 5.0.0 | Consent R4/R5 changes and R5 structure Official versioned definition | Preserve effect/basis/scope/time/status; field renaming is insufficient. | CO3/CO5; R3/R5; L2/L3; P2/P4 |
| 48. PROV5 | Provenance resource: FHIR Release 5, version 5.0.0 | Provenance R4/R5 changes; agent.onBehalfOf Official versioned definition | R5 removes Device/RelatedPerson delegation targets; preserve explicit loss/attestation. | CO2/CO5; R2/R5; L2/L5; P1/P4 |
| 49. AUD5 | AuditEvent resource: FHIR Release 5, version 5.0.0 | AuditEvent R4/R5 changes and outcome structure Official versioned definition | Do not equate technical success with authorization allow. | CO5/CO7; R5/R7; L3/L5; P1/P5 |
| 50. RISK5 | RiskAssessment resource: FHIR Release 5, version 5.0.0 | RiskAssessment R4/R5 changes Official versioned definition | Preserve assessment subject/encounter/output and reason semantics. | CO1/CO6/CO7; R1/R6/R7; L4/Applications; P4/P6 |
| 51. READMIT | Risk prediction models for hospital readmission: a systematic review | Readmission prediction systematic-review conclusions Inherited primary review; no fresh full-text appraisal | Illustrative use-case rationale, not proof of patient benefit. | CO7/CO8; R7/R8; Applications; P6 |
| 52. DECIDE | Reporting guideline for the early-stage clinical evaluation of decision support systems driven by artificial intelligence: DECIDE-AI | DECIDE-AI early clinical evaluation guideline Inherited primary guideline; no fresh full-text appraisal | Future bounded human-factors/clinical evaluation, not results performed here. | CO7/CO8; R7/R8; L5/Applications; P6 |
| XAI-a | DeepXplainer | 10.1016/j.cmpb.2023.107879; full-text appraisal not claimed | Abstract/metadata appraisal; task-specific lung-cancer method does not substantiate the episode contract. | Not retained in main bibliography; S6 records scope decision. |
| XAI-c | XAI-DR-GAN | 10.1016/j.jestch.2026.102438; full-text appraisal not claimed | Publisher-indexed abstract/metadata; retinal grading/explanation does not test clinical boundary control. | Not retained in main bibliography; S6 records scope decision. |
| XAI-d | SE-MMFusionGAN | 10.1007/s44196-026-01428-3; full-text appraisal not claimed | Publisher accepted-version abstract/appendix summaries; image fusion and efficiency are outside the contract test. | Not retained in main bibliography; S6 records scope decision. |
| PROV-origin | Reference 8 | Author project documentation; linked redacted proposal. | 2024 academic problem framing and scope; explicit public-history boundary. | Research provenance only; no independent empirical support for R1-R8 or P1-P6. |
| PROV-code | Reference 9 | Tagged README/SECURITY and release API metadata, read directly. | Bounded synthetic exchange controls, source/release distinctions, archive integrity identifier. | Companion-artifact scope; no complete clinical-AI implementation or proposition evaluation. |
| PROV-poster | Reference 53 | Official 2026 ISAS program text; printed p. 10 and pp. 28-29. | Computer Science Poster 27; title, presenter, venue/date, and published conceptual abstract. | Prior dissemination disclosure; no independently observed attendance or empirical validation. |

# References

1. Health Level Seven International. FHIR Release 4, version 4.0.1: overview. HL7 International. (2019). Available at: https://hl7.org/fhir/R4/overview.html (accessed 10 September 2026).

2. Health Level Seven International. SMART App Launch Implementation Guide, version 2.2.0. HL7 International. (n.d.). Available at: https://hl7.org/fhir/smart-app-launch/STU2.2/ (accessed 10 September 2026).

3. Health Level Seven International. FHIR Bulk Data Access Implementation Guide, version 3.0.0. HL7 International. (n.d.). Available at: https://hl7.org/fhir/uv/bulkdata/STU3/en/index.html (accessed 10 September 2026).

4. Health Level Seven International. CDS Hooks specification, version 2.0.1. HL7 International. (n.d.). Available at: https://cds-hooks.hl7.org/STU2/ (accessed 10 September 2026).

5. Rose S, Borchert O, Mitchell S, Connelly S. Zero trust architecture. NIST Special Publication 800-207. (2020). doi: 10.6028/NIST.SP.800-207.

6. Chandramouli R, Butcher Z. A zero trust architecture model for access control in cloud-native applications in multi-location environments. NIST Special Publication 800-207A. (2023). doi: 10.6028/NIST.SP.800-207A.

7. Tabassi E. Artificial Intelligence Risk Management Framework (AI RMF 1.0). NIST AI 100-1. (2023). doi: 10.6028/NIST.AI.100-1.

8. Oke F. Academic origin and project continuity. In: Consent-Aware FHIR Exchange Prototype, version 1.0.0 [project documentation, with linked privacy-redacted 2024 CS 599 proposal]. GitHub. (2026). Available at: https://github.com/phemmyhorke/consent-aware-fhir-reference-implementation/blob/v1.0.0/ACADEMIC_ORIGIN.md (accessed 10 September 2026).

9. Oke F. Consent-Aware FHIR Exchange Prototype. Version 1.0.0 [software]. GitHub. (2026), released 9 August 2026. Available at: https://github.com/phemmyhorke/consent-aware-fhir-reference-implementation/releases/tag/v1.0.0 (accessed 10 September 2026).

10. Moreau L, Missier P, editors. PROV-DM: the PROV data model. W3C Recommendation. (2013). Available at: https://www.w3.org/TR/prov-dm/ (accessed 10 September 2026).

11. Health Level Seven International. Consent resource: FHIR Release 4, version 4.0.1. HL7 International. (2019). Available at: https://hl7.org/fhir/R4/consent.html (accessed 10 September 2026).

12. Health Level Seven International. Provenance resource: FHIR Release 4, version 4.0.1. HL7 International. (2019). Available at: https://hl7.org/fhir/R4/provenance.html (accessed 10 September 2026).

13. Health Level Seven International. AuditEvent resource: FHIR Release 4, version 4.0.1. HL7 International. (2019). Available at: https://hl7.org/fhir/R4/auditevent.html (accessed 10 September 2026).

14. Health Level Seven International. RiskAssessment resource: FHIR Release 4, version 4.0.1. HL7 International. (2019). Available at: https://hl7.org/fhir/R4/riskassessment.html (accessed 10 September 2026).

15. Williams E, Kienast M, Medawar E, Reinelt J, Merola A, Klopfenstein SAI, et al. A standardized clinical data harmonization pipeline for scalable AI application deployment (FHIR-DHP): validation and usability study. JMIR Med Inform. (2023) 11:e43847. doi: 10.2196/43847.

16. Corbin CK, Maclay R, Acharya A, Mony S, Punnathanam S, Thapa R, et al. DEPLOYR: a technical framework for deploying custom real-time machine learning models into the electronic medical record. J Am Med Inform Assoc. (2023) 30(9):1532-1542. doi: 10.1093/jamia/ocad114.

17. Zhang P, White J, Schmidt DC, Lenz G, Rosenbloom ST. FHIRChain: Applying blockchain to securely and scalably share clinical data. Comput Struct Biotechnol J. (2018) 16:267-278. doi: 10.1016/j.csbj.2018.07.004.

18. Spadacini D. Clinical MLOps: A framework for responsible deployment and observability of AI systems in cloud-native healthcare platforms. Preprints.org [preprint, version 1]. (2026). Posted February 28, 2026. Available at: https://www.preprints.org/manuscript/202602.1951 [Accessed September 10, 2026]. The repository links a journal version, doi: 10.56147/aaiet.2.1.120; the comparison here uses the accessible preprint.

19. Maimaitiaili M, Jiamaliding Y, Dai G, Xiao H, Kuerbanjiang W, Yi Y. Artificial intelligence platform architecture for hospital systems: systematic review. J Med Internet Res. (2025) 27:e79788. doi: 10.2196/79788.

20. Ayaz M, Pasha MF, Alzahrani MY, Budiarto R, Stiawan D. The Fast Health Interoperability Resources (FHIR) standard: systematic literature review of implementations, applications, challenges and opportunities. JMIR Med Inform. (2021) 9(7):e21929. doi: 10.2196/21929.

21. Balch JA, Ruppert MM, Loftus TJ, Guan Z, Ren Y, Upchurch GR Jr, et al. Machine learning-enabled clinical information systems using Fast Healthcare Interoperability Resources data standards: scoping review. JMIR Med Inform. (2023) 11:e48297. doi: 10.2196/48297.

22. Strasberg HR, Rhodes B, Del Fiol G, Jenders RA, Haug PJ, Kawamoto K. Contemporary clinical decision support standards using Health Level Seven International Fast Healthcare Interoperability Resources. J Am Med Inform Assoc. (2021) 28(8):1796-1806. doi: 10.1093/jamia/ocab070.

23. Kent K, Souppaya M. Guide to computer security log management. NIST Special Publication 800-92. (2006). doi: 10.6028/NIST.SP.800-92.

24. Park J, Sandhu R. The UCONABC usage control model. ACM Trans Inf Syst Secur. (2004) 7(1):128-174. doi: 10.1145/984334.984339.

25. Torres-Arias S, Afzali H, Kuppusamy TK, Curtmola R, Cappos J. in-toto: Providing farm-to-table guarantees for bits and bytes. In: 28th USENIX Security Symposium. Berkeley, CA: USENIX Association. (2019). p. 1393-1410. Available at: https://www.usenix.org/conference/usenixsecurity19/presentation/torres-arias [Accessed September 10, 2026].

26. Paterson C, Hawkins R, Picardi C, Jia Y, Calinescu R, Habli I. Safety assurance of Machine Learning for autonomous systems. Reliab Eng Syst Saf. (2025) 264:111311. doi: 10.1016/j.ress.2025.111311.

27. World Wide Web Consortium. Trace Context, version 1. W3C Recommendation. (2021). Available at: https://www.w3.org/TR/trace-context/ (accessed 10 September 2026).

28. Mitchell M, Wu S, Zaldivar A, Barnes P, Vasserman L, Hutchinson B, et al. Model cards for model reporting. In: Proceedings of the Conference on Fairness, Accountability, and Transparency. (2019) New York, NY: ACM. p. 220-229. doi: 10.1145/3287560.3287596.

29. Wiens J, Saria S, Sendak M, Ghassemi M, Liu VX, Doshi-Velez F, et al. Do no harm: a roadmap for responsible machine learning for health care. Nat Med. (2019) 25(9):1337-1340. doi: 10.1038/s41591-019-0548-6.

30. Lekadir K, Frangi AF, Porras AR, Glocker B, Cintas C, Langlotz CP, et al. FUTURE-AI: international consensus guideline for trustworthy and deployable artificial intelligence in healthcare. BMJ. (2025) 388:e081554. doi: 10.1136/bmj-2024-081554.

31. Lee JD, See KA. Trust in automation: designing for appropriate reliance. Hum Factors. (2004) 46(1):50-80. doi: 10.1518/hfes.46.1.50_30392.

32. Gaube S, Suresh H, Raue M, Merritt A, Berkowitz SJ, Lermer E, et al. Do as AI say: susceptibility in deployment of clinical decision-aids. NPJ Digit Med. (2021) 4:31. doi: 10.1038/s41746-021-00385-9.

33. Wani NA, Kumar R, Mamta, Bedi J, Rida I. Explainable AI-driven IoMT fusion: Unravelling techniques, opportunities, and challenges with Explainable AI in healthcare. Inf Fusion. (2024) 110:102472. doi: 10.1016/j.inffus.2024.102472.

34. Weiskopf NG, Weng C. Methods and dimensions of electronic health record data quality assessment: enabling reuse for clinical research. J Am Med Inform Assoc. (2013) 20(1):144-151. doi: 10.1136/amiajnl-2011-000681.

35. US Department of Health and Human Services. Summary of the HIPAA Security Rule. HHS. (n.d.). Available at: https://www.hhs.gov/hipaa/for-professionals/security/laws-regulations/index.html (accessed 10 September 2026).

36. Davis SE, Lasko TA, Chen G, Siew ED, Matheny ME. Calibration drift in regression and machine learning models for acute kidney injury. J Am Med Inform Assoc. (2017) 24(6):1052-1061. doi: 10.1093/jamia/ocx030.

37. Sittig DF, Singh H. A new sociotechnical model for studying health information technology in complex adaptive healthcare systems. Qual Saf Health Care. (2010) 19 Suppl 3:i68-i74. doi: 10.1136/qshc.2010.042085.

38. Joint Task Force Transformation Initiative. Guide for conducting risk assessments. NIST Special Publication 800-30 Revision 1. (2012). doi: 10.6028/NIST.SP.800-30r1.

39. US Department of Health and Human Services. What is the difference between consent and authorization under the HIPAA Privacy Rule?. HHS. (n.d.). Available at: https://www.hhs.gov/hipaa/for-professionals/faq/264/what-is-the-difference-between-consent-and-authorization/index.html (accessed 10 September 2026).

40. US Department of Health and Human Services. Research. HHS. (n.d.). Available at: https://www.hhs.gov/hipaa/for-professionals/special-topics/research/index.html (accessed 10 September 2026).

41. US Department of Health and Human Services. Minimum necessary. HHS. (n.d.). Available at: https://www.hhs.gov/hipaa/for-professionals/privacy/guidance/minimum-necessary-requirement/index.html (accessed 10 September 2026).

42. US Department of Health and Human Services. Guidance regarding methods for de-identification of protected health information in accordance with the HIPAA Privacy Rule. HHS. (n.d.). Available at: https://www.hhs.gov/hipaa/for-professionals/special-topics/de-identification/index.html (accessed 10 September 2026).

43. Health Level Seven International. US Core Implementation Guide, version 9.0.0 (STU 9). HL7 International. (2026). Available at: https://hl7.org/fhir/us/core/STU9/ (accessed 10 September 2026).

44. Health Level Seven International. DocumentReference resource: FHIR Release 4, version 4.0.1. HL7 International. (2019). Available at: https://hl7.org/fhir/R4/documentreference.html (accessed 10 September 2026).

45. Health Level Seven International. FHIR Release 4, version 4.0.1: RESTful API. HL7 International. (2019). Available at: https://hl7.org/fhir/R4/http.html (accessed 10 September 2026).

46. Health Level Seven International. FHIR Release 4, version 4.0.1: Attachment detailed definitions. HL7 International. (2019). Available at: https://hl7.org/fhir/R4/datatypes-definitions.html#Attachment.hash (accessed 10 September 2026).

47. Health Level Seven International. Consent resource: FHIR Release 5, version 5.0.0. HL7 International. (2023). Available at: https://hl7.org/fhir/R5/consent.html (accessed 10 September 2026).

48. Health Level Seven International. Provenance resource: FHIR Release 5, version 5.0.0. HL7 International. (2023). Available at: https://hl7.org/fhir/R5/provenance.html (accessed 10 September 2026).

49. Health Level Seven International. AuditEvent resource: FHIR Release 5, version 5.0.0. HL7 International. (2023). Available at: https://hl7.org/fhir/R5/auditevent.html (accessed 10 September 2026).

50. Health Level Seven International. RiskAssessment resource: FHIR Release 5, version 5.0.0. HL7 International. (2023). Available at: https://hl7.org/fhir/R5/riskassessment.html (accessed 10 September 2026).

51. Kansagara D, Englander H, Salanitro A, Kagen D, Theobald C, Freeman M, et al. Risk prediction models for hospital readmission: a systematic review. JAMA. (2011) 306(15):1688-1698. doi: 10.1001/jama.2011.1515.

52. Vasey B, Nagendran M, Campbell B, Clifton DA, Collins GS, Denaxas S, et al. Reporting guideline for the early-stage clinical evaluation of decision support systems driven by artificial intelligence: DECIDE-AI. Nat Med. (2022) 28(5):924-933. doi: 10.1038/s41591-022-01772-9.

53. Oke F. Operationalizing AI on Secure FHIR Pipelines: A Zero-Trust Cloud Reference Architecture for EHR Interoperability [conference abstract, Computer Science Poster 27]. In: 118th Annual Meeting of the Illinois State Academy of Science, 18 April 2026, Peoria, Illinois. Program supplement to Transactions of the Illinois State Academy of Science. (2026) 118(Suppl):28-29. Available at: https://myisas.org/wp-content/uploads/2026/05/Annual-Meeting-Program-2026-FINAL.pdf (accessed 10 September 2026).
